# Global genomic surveillance uncovers emergence and spatiotemporal patterns of World Health Organization priority antibiotic resistance in invasive *Salmonella*

**DOI:** 10.1038/s41467-026-76354-1

**Published:** 2026-08-18

**Authors:** Yuhang Pei, Ziru Yang, Xiaolu Pang, Wufuer. Xiayidan, Meilian Huang, Jinhua Meng, Yufeng Qiu, Mingliu Wang, Hongxia Yang, Chunmei Jing, Mei Zeng, Yue Liu, Wenqing Wang, Yibin Zhou, Sufang Ma, Na Lyu, Mengqi Qu, Chaochao Zhao, Jiangtao Wang, Jiale Chen, Baoli Zhu, George F. Gao, Xuebin Xu, Yanan Wang

**Affiliations:** 1 International Joint Research Center of National Animal Immunology, College of Veterinary Medicine, Henan Agricultural University, Zhengzhou, China; 2 Longhu Laboratory of Advanced Immunology, Zhengzhou, China; 3 Department of Clinical Laboratory, The Fourth People’s Hospital of Nanning, Nanning, China; 4 Department of Microbiology, Xinjiang Uygur Autonomous Region Center for Disease Control and Prevention, Urumqi, China; 5 Department of Laboratory Medicine, Children’s Hospital of Fudan University (Xiamen Branch), Xiamen Children’s Hospital, Xiamen, China; 6 Center of Clinical Medical Laboratory, Shanxi Children’s Hospital, Taiyuan, China; 7 Fujian Center for Disease Control and Prevention, Fujian Provincial Key Laboratory of Zoonosis Disease Research, Fuzhou, China; 8 Guangxi Zhuang Autonomous Region Center for Disease Control and Prevention, Nanning, China; 9 Shanxi Center for Disease Control and Prevention, Taiyuan, China; 10 Children’s Hospital of Chongqing Medical University, Chongqing, China; 11 Department of Infectious Diseases, Children’s Hospital of Fudan University, Shanghai, China; 12 Division of Pathogen Testing and Analysis, Shanghai Municipal Center for Disease Control and Prevention, Shanghai, China; 13 Department of Microbiology, Shanghai Pudong New Area Center for Disease Control and Prevention (Shanghai Pudong New Area Health Supervision Institute), Shanghai, China; 14 Department of Infectious Disease Control, Center for Disease Control and Prevention of Minhang District, Shanghai, China; 15 Beijing Key Laboratory of Antimicrobial-Resistant Pathogen Microbiology and AI-Empowered Containment, Institute of Microbiology, Chinese Academy of Sciences, Beijing, China; 16 University of Chinese Academy of Sciences, Beijing, China; 17 Chinese Center for Disease Control and Prevention (China CDC), Beijing, China

**Keywords:** Bacterial infection, Data mining, Policy and public health in microbiology, Clinical microbiology

## Abstract

Antimicrobial resistance (AMR) in invasive *Salmonella* infections remains a global public health concern, and enhanced high-quality genomic surveillance is essential to inform guidelines and policies. Here, we aimed to describe clinically key antibiotic resistance trends in invasive *Salmonella* infections using 7691 isolates derived from national surveillance and publicly available datasets between 1954 and 2024. 29 (37.7%) of 77 countries identified at least one isolate with resistance or decreased susceptibility to ciprofloxacin, 25 (32.5%) and 29 (37.7%) countries resistance or decreased susceptibility to ceftriaxone and cefixime, 8 (10.4%) countries resistance to azithromycin, 15 (19.5%) countries resistance to fosfomycin, 10 (13.0%) countries resistance to colistin. Overall, 7.39% of 7691 isolates were resistant or decreased susceptibility to ciprofloxacin, 7.91% reported resistance or decreased susceptibility to 3GCs, 4.23% reported resistance to fosfomycin, and 0.79% reported resistance to azithromycin. Globally, azithromycin resistance is increasing, as is resistance or decreased susceptibility to ciprofloxacin, 3GCs, and 4GCs. However, surveillance levels remain inadequate in African and South American regions. We generated a global map of clinically key antibiotic resistance genes in invasive *Salmonella* infections worldwide. This retrospective, global, longitudinal genomic epidemiology study on invasive *Salmonella* provides evidence-based data for clinical guidelines, genomic surveillance, AMR control, and public health policies.

## Abstract (web summary)

Antimicrobial resistance in invasive *Salmonella* is an escalating global health threat, yet its global trends and genomic evidence remain poorly understood. Wang and Xu *et al*. use a state-of-the-art pipeline integrating global genomics to reveal emergence and spatiotemporal patterns of WHO priority antibiotic resistance in invasive *Salmonella*.

## Introduction

*Salmonella enterica* (*S*. *enterica*) is a leading cause of diarrheal infections and a key pathogen of invasive infections (usually bloodstream infections, BSIs) worldwide^1,2^. According to the estimates of the Global Burden of Disease Study (GBDS)^3,4^, *S*. *enterica* subspecies *enterica* serovar Typhi (*S*. Typhi) and *S*. Paratyphi infections led to about 205,300 deaths worldwide, and invasive non-typhoidal *Salmonella* (iNTS) infections led to about 650,000 deaths yearly worldwide. In China, it was estimated that 1173 deaths were attributed to blood-invasive *S*. *enterica* infections in 2019^5^. Due to the emergence of antimicrobial resistance (AMR), invasive infections (usually BSIs) caused by Gram-negative bacteria have become one of the leading causes of death from BSIs worldwide^6^. Globally, *S*. *enterica* was one of the most frequently isolated pathogens from invasive infections^7–10^. Hence, invasive infections remain a global clinical and public health problem.

Fluoroquinolones (ciprofloxacin), third-generation cephalosporins (3GCs, ceftriaxone), and macrolides (azithromycin) were recommended as the first or second choice for treating invasive *Salmonella* infections. However, fluoroquinolone-resistant *S*. *enterica* has been listed in the 2024 World Health Organization Bacterial Priority Pathogens List (WHO BPPL) by WHO, owing to the emergence of AMR (especially to fluoroquinolones, cephalosporins, and carbapenems)^11,12^. Investigation of epidemiology, burden, and drivers of infections by drug-resistant typhoid and NTS (especially those resistant to cephalosporins and fluoroquinolones) across socioeconomic settings has been listed in 40 research priorities for AMR in human health^13^. In 2021, it was estimated that 4.71 million deaths were associated with AMR infections, 1.14 million deaths were attributed to AMR bacterial infections worldwide^14^, and 160,268 deaths were attributable to AMR in China^15^. The emergence and spread of *S*. *enterica* conferring resistance to clinically critical antibiotics, including first- and second-line antibiotics, and even last-line antibiotics, has restricted our options for treatment, underscoring the urgency of genomic surveillance.

Previous studies reported that the number of mobile antibiotic resistance genes (ARGs) per isolate increased 1.8 and 2.7 times in both human and non-human origins in China, and also identified some isolates carrying mobile colistin resistance genes (for example, *mcr-1*, *mcr-3*, and *mcr-9*) and tigecycline resistance gene *tet*(X4) by analyzing the Chinese local passive surveillance system for *Salmonella* database^16–18^. Moreover, the emergence and spread of azithromycin-resistant *Salmonella* strains, mainly caused by *mph(A)*, poses an important threat to public health^19–24^. Zheng et al. reported that the prevalence of azithromycin resistance rates was higher in invasive *S*. Enteritidis isolates (5.3%) than in sporadic isolates (1.8%) and outbreak isolates (1.69%) in China^25^. Globally, by analyzing over 208 thousand *Salmonella* genomes across 100 years, our recent work revealed alarming increases in AMR prevalence in the majority of antibiotic classes with substantial geographic, serovar-related, and source-specific variations^22^ and evidenced that WGS surveillance provided future directions for integrating genomic monitoring into combating AMR and informing outbreak responses^26–31^.

Due to the emergence and dissemination of mobile fosfomycin resistance genes and limited options for treating carbapenem-resistant *Enterobacteriaceae* infections, fosfomycin has been moved up to the highest priority critically important antimicrobials by WHO^32^. Recent studies revealed the geographical distribution, serovar diversity, and the prevalence of fosfomycin resistance genes in *S*. Typhimurium isolates with a distinct rough colony morphology^16,21,22,33,34^. In recent years, a few studies have investigated the AMR patterns and genomic features of invasive *S*. *enterica* using a small dataset in a limited region using phenotypic or genomic methods^16,35,36^. However, no studies focused on the prevalence and spatiotemporal trends of ciprofloxacin and 3GCs-resistant invasive *S*. *enterica* causing invasive infections using nationwide, longitudinal genomic epidemiology data in China, as well as on a global scale.

To address this knowledge gap, we first used a total of 910 invasive *S*. *enterica* isolates collected from nationwide passive surveillance in the Chinese local passive surveillance system for *Salmonella* database during 1994 and 2023, combined with 998 publicly available genomes, to investigate the dynamic prevalence of ESBL (3GCs/4GCs), ciprofloxacin, azithromycin, and fosfomycin resistance. Then, we assembled a global invasive *S*. *enterica* genomic dataset (*n* = 7691) to uncover the spatiotemporal trends of clinically critical antibiotic resistance and explore the driving force of horizontal gene transfer of AMR with high resolution. Utilizing data from the Chinese local passive surveillance system for the *Salmonella* database and public data, this study aims to provide a comprehensive atlas of invasive *S*. *enterica* conferring to clinically critical antibiotics over the past seven decades worldwide, to provide in-depth insights for AMR control and policy-making, and guide clinics and veterinary medicine.

## Results

### Construction of a global invasive *Salmonella* genomic dataset

In this study, the invasive *Salmonella* data used here included two parts. One part is from the retrospective genomic epidemiological study in China, and another part is from publicly available *Salmonella* genomes in the National Center of Biotechnology Information (NCBI). A total of 585,220 publicly available *Salmonella* genomes were downloaded from the National Center for Biotechnology Information (as of February 14, 2025), after screening by using SeqSero2, SISTR, and MLST, as well as clear metadata information. A total of 260,195 high-quality genomes were used for the following analysis. Among them, 117,461 strains were isolated from humans, and 6781 strains were isolated from blood, urine, and others from extra-intestinal infection sites, and were further selected for AMR analysis.

**Fig. 1:**
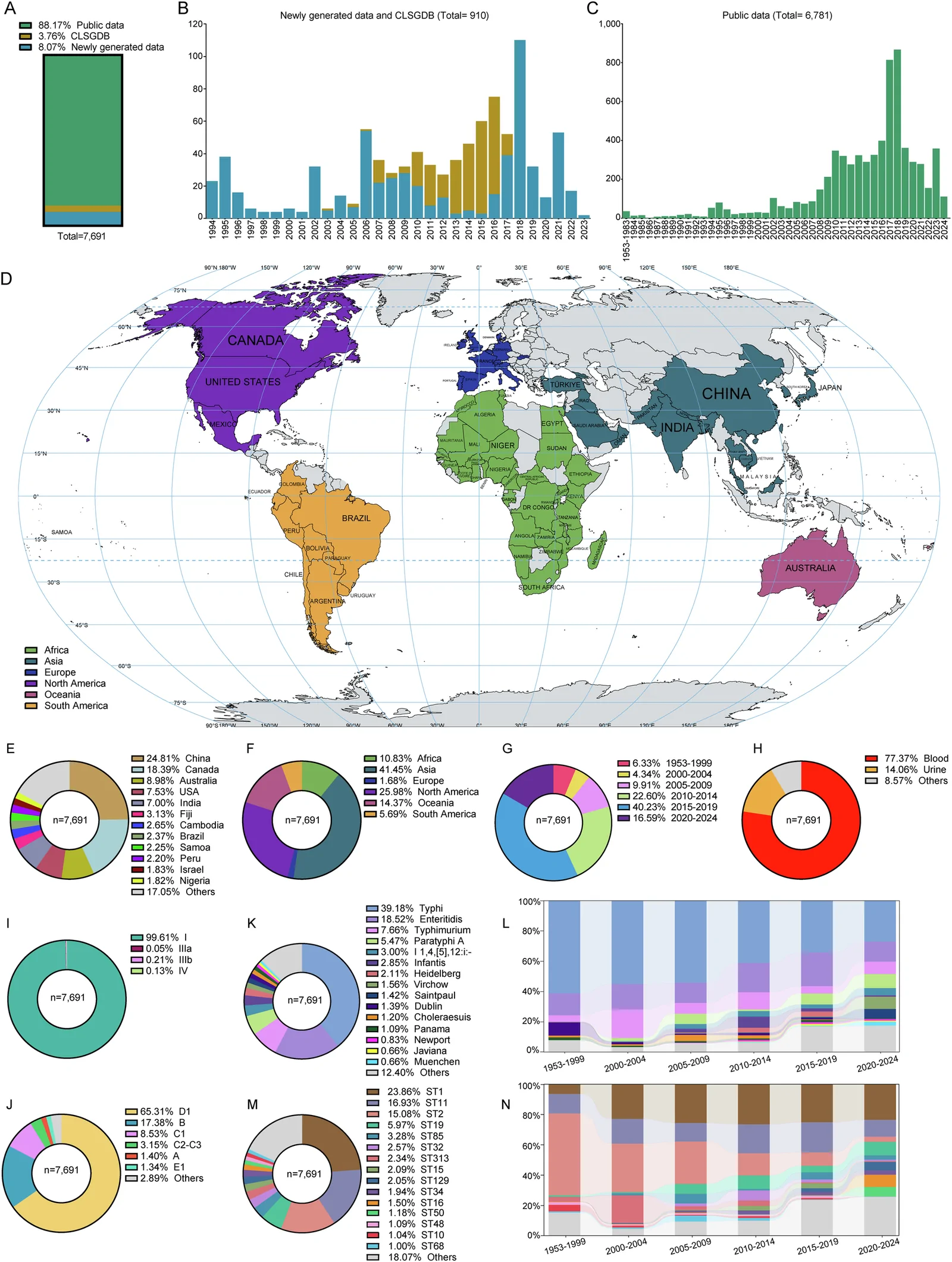
Genomic collection and geographical distribution of invasive *Salmonella* isolates used to construct the invasive *Salmonella* infections population structure. **A** Total number of genomes per collection. Stacked color bars are proportional to the number of genomes from that source. A total of 910 newly generated WGS data were included in this study. The CLSGDB means: the Chinese Local *Salmonella* Genome DataBase (https://nmdc.cn/clsgdbv2/). **B** The number of public genomic data from different years. **C** The number of newly generated WGS data in this study in different years. **D** Geographical distribution of invasive *Salmonella* isolates in 77 countries. The global map was built using R v4.5.2. **E** The proportion of isolates in the top 12 countries. **F** The proportion of *Salmonella* isolates in six continents. **G** The proportion of *Salmonella* isolates among different time periods. **H** The proportion of isolates from different infection sites. **I** The proportion of *Salmonella* isolates from different subspecies. **J** The proportion of *Salmonella* isolates in the top six serogroups. **K** The proportion of *Salmonella* isolates in the top 15 serovars. **L** Temporal trends of top 15 serovars. The color annotation of different serovars is consistent with Fig. 1K. **M** The proportion of isolates in the top 15 STs. **N** Temporal trends of top 15 STs. The color annotation of different serovars is consistent with Fig. 1M. Source data are provided as a Source Data file.

The global invasive *Salmonella* collection analyzed in this study (*n *= 7691) (Fig. 1A, Supplementary Data 1, 2) included 621 (8.07%) isolates (collected between 1994 and 2023) from the six Chinese provincial-level administrative areas (PLAs), 289 (3.76%) isolates (Fig. 1B) from our previous study in the CLSGDBv2 (https://nmdc.cn/clsgdbv2)^16,21^, and 6781 (88.17%) public contextual collections (collected between 1953 and 2024) (Fig. 1C) from the NCBI^22^. We sequenced the genomes of 621 non-repetitive blood-invasive *S*. *enterica* isolates covering 48 serovars from 6 PLAs’ comprehensive passive surveillance dataset (including Shanghai Municipality, Guangxi Zhuang Autonomous Region, Fujian Province, Shanxi Province, Xinjiang Uygur Autonomous Region, and Chongqing Municipality) registered in the CLSSSDB. The global collection represents 6 inhabited continents and includes isolates distributed in 77 countries collected between 1953 and 2024 (Fig. 1D). A detailed metadata description of 7691 isolates is provided in Supplementary Data 1, 2.

Overall, the number of invasive isolates collected in China was the highest (24.81%), followed by Canada (18.39%), Australia (7.53%), and India (7.00%) (Fig. 1E, Supplementary Fig. 1). Among these, 41.45% were isolated from Asia, followed by North America (25.98%), Oceania (14.37%), and Africa (10.83%) (Fig. 1F). These genomes were chronologically divided into 6 period groups, and the majority (79.4%) were isolated in the past 15 years (Fig. 1G). A total of 5951 (77.37%) of these invasive isolates were isolated from bloodstream infections, 14.06% were derived from urinary tract infections, and 8.57% were taken from other body sites (Fig. 1H). In this global collection, 99.61% belonged to subspecies I (*enterica*) (Fig. 1I) and 65.31% belonged to serogroup D1 (Fig. 1J). A total of 148 serovars were identified; the most dominant serovar was *S*. Typhi (39.18%) (especially in Asia, Oceania, and South America), followed by Enteritidis and Typhimurium (Fig. 1K, Supplementary Fig. 2A). Regional differences in the prevalence of serovars in six continents were observed (Supplementary Fig. 2A). Globally, we observed an obvious shift in the prevalence of serovars causing invasive infections. For example, the prevalence of *S*. Typhi, *S*. Choleraesuis, and *S*. Infantis showed declining trends, while *S*. Paratyphi A and several NTS serovars (for example, *S*. I 1,4,[5],12:i:-, *S*. Virchow, *S*. Saintpaul, and *S*. Muenchen) showed increasing trends (Fig. 1L, Supplementary Fig. 2B).

Genotyping scheme analysis indicated that *S*. Typhi genotype 4.3.1.2.1 and *S*. Paratyphi A genotypes 1, 2.3.2, and 2.4.7 were identified in the Chinese mainland for the first time. The dominant *S*. Typhi genotype in Asia was genotype 4 (4.3), the dominant genotypes in Oceania were genotype 4 (4.2) and genotype 3 (3.5), the dominant genotype in North America was genotype 4 (4.3), and the dominant genotype in Africa was genotype 3 (3.1) (Supplementary Fig. 3A, B). The dominant genotypes in China were genotype 2 (2.1) and genotype 3 (3.2) (Supplementary Fig. 3C, D). Since 2007, genotype 4 has gradually become the dominant genotype worldwide, while after 2013, genotype 4.3 has gradually become dominant (Supplementary Fig. 3E, F). In Africa, genotype 4.3 became the dominant genotype since 2013, and in Asia, 4.3 became the dominant genotype after 2005. In invasive *S*. Paratyphi A, genotype 2.3 (2.3.3) was prevalent in Asia, while genotype 2.4 accounts for above 50% in Oceania and North America (Supplementary Fig. 4A, B). In China, genotype 2.3 (2.3.3) accounts for over 90% of cases, while genotype 2.4 accounts for over 50% in Australia, India, and Canada. In Israel, all genotypes were 2.4, mainly 2.4.2 and 2.4.8 (Supplementary Fig. 4C, D). Globally, the prevalence of genotype 2.3 is gradually declining, driven by a decrease in the proportion of 2.3.3, while the proportions of 2.3.2 and 2.3.4 are on the rise (Supplementary Fig. 4E, F). The proportion of genotype 2.4 showed an increasing trend, primarily driven by the rise of subtypes 2.4.1, 2.4.2, 2.4.3, 2.4.5, and 2.4.8.

To investigate the population structure, a minimum spanning tree (MST) was constructed, and multilocus sequence typing (MLST) analysis assigned these invasive *S*. *enterica* isolates to 315 STs (Supplementary Fig. 5), including two newly discovered STs, *S*. Paratyphi A ST10417 and *S*. Enteritidis ST10419, which were discovered in China for the first time. The most dominant ST was *S*. Typhi ST1 (23.86%), followed by *S*. Enteritidis ST11, *S*. Typhi ST2, and ST19 (*S*. Typhimurium and *S*. I 1,4,[5],12:i:-) (Fig. 1M, Supplementary Fig. 5A). Notably, we observed that the prevalence of *S*. Typhi ST2, *S*. Typhimurium ST313, and *S*. Choleraesuis ST68 showed declining trends, while increasing trends in the prevalence of ST19 and ST34 (*S*. Typhimurium and *S*. I 1,4,[5],12:i:-), *S*. Paratyphi A ST129 and ST85 (*S*. Paratyphi A), *S*. Virchow ST16, and *S*. Saintpaul ST50 (Fig. 1N, Supplementary Fig. 5B). The dominant STs in Asia and Oceania, Africa, North America, Europe, and South America are *S*. Typhi ST1, *S*. Enteritidis ST11, *S*. Kentucky ST198, and *S*. Infantis ST32, respectively (Supplementary Fig. 6A). In Europe, the proportion of the dominant sequence type, *S*. Kentucky ST198, has been declining year by year since 2009 (Supplementary Fig. 6B). Overall, we assembled a global invasive *Salmonella* genomic catalog and expanded our insight into the diversity and predominant clone switch during 1953–2024.

### Spatiotemporal dynamics of clinically critical antibiotic resistance markers in invasive *Salmonella* worldwide

**Fig. 2:**
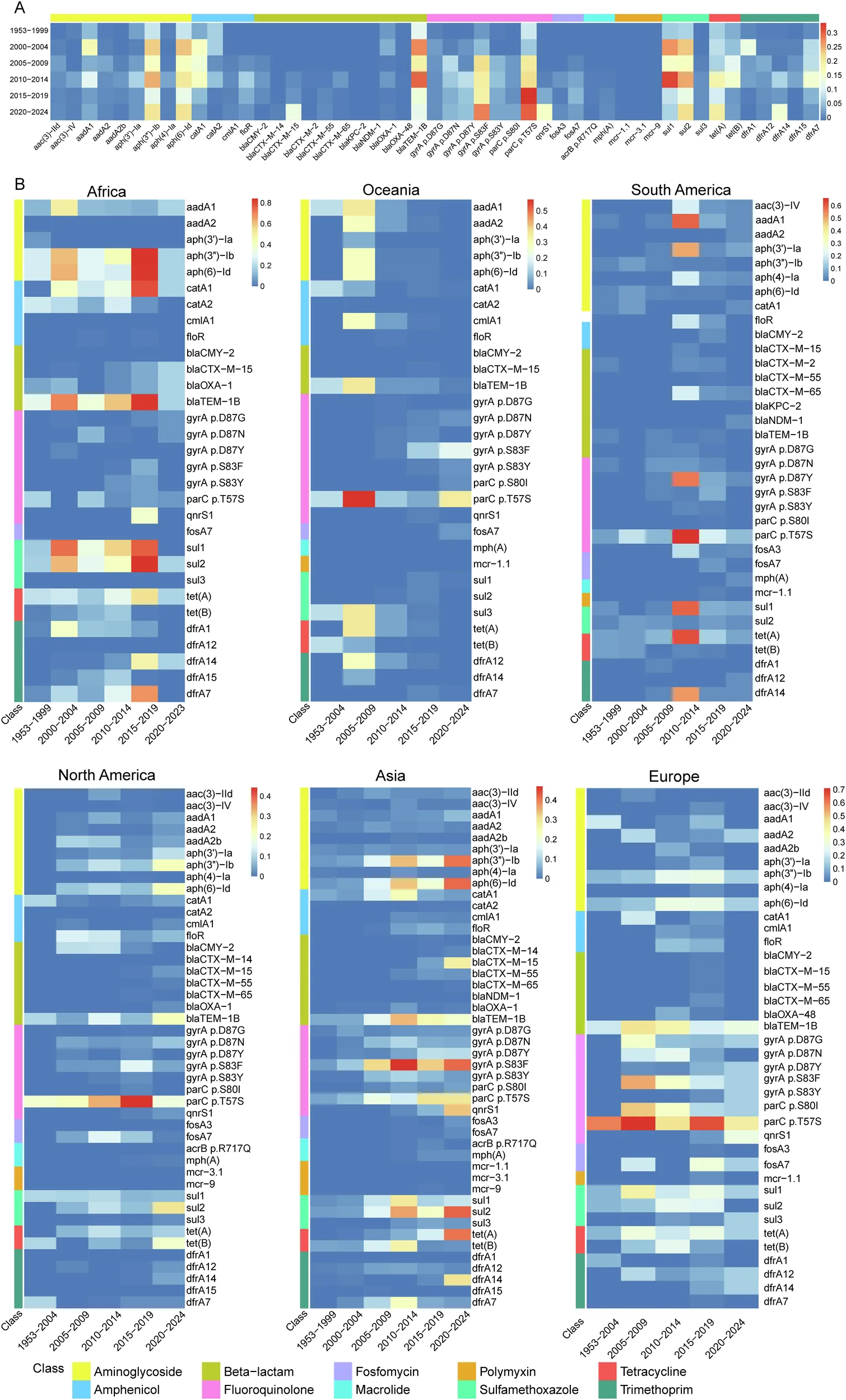
Temporal dynamics of clinical key antibiotic resistance genes in *S*. *enterica* causing human invasive infections. **A** Temporal trends of clinical key ARGs in global invasive *Salmonella* causing human invasive infections. **B** Temporal trends of clinical key ARGs in six continents. ARGs greater than 1%, as well as ARGs conferring resistance to clinically critical antibiotics, including 3GCs, 4GCs, fosfomycin, carbapenems, polymyxins, and azithromycin, etc. The different colors of ARGs correspond to the colors of different types of antibiotics. Source data are provided as a Source Data file.

Based on WGS prediction, we mapped the global ARG profiles of invasive *Salmonella* worldwide. A total of 199 ARGs were predicted, which confer resistance to 13 types of antimicrobial agents and disinfectants (Supplementary Data 3). We investigated *bla*_CTX-M_-encoding genes to be markers for ESBL-production and detected 16 *bla*_CTX-M_ variants in 445 (5.79%) of 7691 isolates. We detected a significant increase in the prevalence of *bla*_CTX-M_-positive isolates from 1953–1999 (one [0.21%] of 487 isolates) to 2020–2024 periods (210 [16.46%] of 1276 isolates). *bla*_CTX-M-15_ (3.48%) was the most dominant *bla*_CTX-M-15_ type, followed by *bla*_CTX-M-55_ (0.88%), *bla*_CTX-M-65_ (0.82%) (Supplementary Data 4). We observed increased prevalence in the number of *bla*_CTX-M-15_, *bla*_CTX-M-55_, and *bla*_CTX-M-14_ worldwide (Fig. 2A, Supplementary Data 4), especially in North America, Asia, and Africa (Fig. 2B, Supplementary Data 4). Among the top 16 serovars, we observed that *bla*_CTX-M-15_-positive isolates increased significantly in *S*. Typhi, *bla*_CTX-M-55_-positive isolates increased significantly in *S*. Choleraesuis and *S*. Kentucky (Supplementary Fig. 7, Supplementary Data 5). Among the top 16 serovar STs, *bla*_CTX-M-15_-positive isolates increased significantly in *S*. Typhi ST1 and *S*. Typhimurium ST313, and *bla*_CTX-M-55_-positive isolates increased significantly in *S*. Kentucky ST198 (Supplementary Fig. 8, Supplementary Data 6). *bla*_CTX-M-14_-positive isolates increased significantly in *S*. Enteritidis ST11.

Fluoroquinolone nonsusceptibility was observed in 4085 (53.11%) of 7691 isolates. There was a significant increase from 71 (14.58%) of 487 isolates during the 1953–1999 period to 790 (61.91%) of 1276 isolates during the 2020–24 period. Only 416 (10.18%) of 4085 fluoroquinolone non-susceptible isolates were also *bla*_CTX-M_-positive, whereas most (416 [93.48%] of 445 isolates) of *bla*_CTX-M_-positive isolates were also fluoroquinolone non-susceptible. The prevalence of acquired gene *qnrS1* and chromosomal gene mutation *parC* p.T57S and *parC* p.S80R increased (Fig. 2A), especially in North America and Asia (Fig. 2B). We observed that the prevalence of chromosomal gene mutations *gyrA* p.S83F, *gyrA* p.S83Y, and *gyrA* p.D87N increased in Oceania (Fig. 2B). Notably, some dominant serovars, including *S*. Typhi, *S*. Enteritidis, *S*. Typhimurium, *S*. Infantis, and *S*. Kentucky, made the largest contribution to the increase in *qnrS1* in the collection, which were mainly distributed in *S*. Typhi ST1, *S*. Enteritidis ST11, *S*. Typhimurium ST19, *S*. Infantis ST32, *S*. I 1,4,[5],12:i:- ST34, *S*. Panama ST48, and *S*. Kentucky ST198 (Supplementary Fig. 8). The number of *gyrA* p.S83F showed an increasing trend in *S*. Typhi (ST1 and ST2), and *S*. Infantis ST32, while decreasing in *S*. Paratyphi A ST85.

We identified 322 (7.88%) of 4085 fluoroquinolone non-susceptible isolates that were also fosfomycin resistance gene *fosA*-positive. The prevalence of *fosA7* increased in Oceania and Asia, while *fosA3* increased mainly in *S*. Enteritidis ST11 and *S*. Kentucky ST198 from Asia (Fig. 2B). We also observed that 50 (1.22%) of 4085 fluoroquinolone non-susceptible isolates were also azithromycin resistance gene *mph(A)*-positive. There was a significant increase from 5 (0.55%) of 914 isolates during the 2010–2014 period to 18 (2.28%) of 790 isolates during the 2020–24, especially in North America, South America, and Asia (Fig. 2A, B). The significantly increasing trend was mainly identified in *S*. Kentucky ST198.

Regional disparities in the geographical distribution of AMR markers across six continents were observed (Supplementary Data 7). For example, *bla*_CTX-M-65_ (46 [10.5%] of 438 isolates) was the most common in South America, and both *bla*_CTX-M-15_ (214 [6.71%] of 3,188 isolates) and *bla*_CTX-M-55_ (63 [1.98%] of 3188 isolates) were the most common in Asia (Supplementary Fig. 9A). *bla*_CTX-M-15_ was the most common in *S*. Typhi, *bla*_CTX-M-65_ was the most common in *S*. Infantis, while *bla*_CTX-M-55_ and *bla*_CTX-M-14_ was the most common in *S*. I 1,4,[5],12:i:- (Supplementary Fig. 9B). It was noteworthy that 8 and 20 isolates carried carbapenem resistance genes (*bla*_KPC-2_ [*n* = 1], *bla*_NDM-1_ [*n* = 3], and *bla*_OXA-48_ [*n* = 4]) and colistin resistance genes (*mcr-1.1* [*n* = 9], *mcr-3.1* [*n* = 4], and *mcr-9* [*n* = 7]), respectively. All carbapenemase-producing isolates were also fluoroquinolone non-susceptible. Except for three *mcr-1.1*-positive isolates, the other 17 *mcr-*positive isolates were also fluoroquinolone non-susceptible.

### Regional disparities of *Salmonella* conferring resistance to clinically critical antibiotics across geographic locations and predominant clones

In this study, the resistance phenotypes of all isolates were predicted based on the presence of AMR genotypes. Regional disparities of invasive *Salmonella* conferring resistance to clinically critical antibiotics were observed (Supplementary Data 8). A total of 70 antimicrobial classes were analyzed; only clinically critical antibiotics were described in this study. Among these 3GC-resistant isolates, 7.91%, 7.91%, 6.36%, and 0.05% of 7691 invasive isolates were resistant to cefotaxime, ceftazidime, ceftriaxone, and cefixime, respectively (Table 1 and Supplementary Data 8). Moreover, 7.27% of these isolates were resistant to 4GC (for example, cefepime). Among these 3GC-resistant isolates, the highest rate was in South America, followed by Europe and Asia (Table 1). Hotspots of ESBL-producing *Salmonella* were Brazil (8.24%), Switzerland (8.20%), and China (7.02%). Only 0.10%, 0.10%, and 0.05% were resistant to meropenem, imipenem, and ertapenem, respectively. Hotspots of carbapenem-resistant were Switzerland (4.92%). The majority of carbapenem-resistant strains were identified in *S*. Javiana, meropenem and imipenem-resistant isolates were mainly identified in Europe, while ertapenem was identified in South America. Although differences in the resistance of the 3GCs and 4GCs among different serovars and STs, the highest resistance rate was in *S*. Infantis ST32. 32.22% and 18.33% of ST313 strains were resistant to 4GCs (cefepime) and 3GCs (cefotaxime and ceftazidime), respectively. Notably, of the 7.63% ESBL-producing *S*. Typhi, 12.43% of them were ST1; only 0.17% of ST2 were ESBL-producing isolates.

**Table 1:** Differences in clinically critical antibiotic resistance in invasive *Salmonella* across geographic locations, sources, serovars, and STs

Category		3GCs		4GC	Carbapenem		Fosfomycin	Macrolide	Polymyxin	Fluoroquinolone	MDR
		Cefotaxime/ Ceftazidime	Ceftriaxone	Cefepime	Ertapenem	Imipenem/Meropenem	Fosfomycin	Azithromycin	Colistin	Ciprofloxacin	
**Continent**	**Global (7691)**	7.91%	6.36%	7.27%	0.05%	0.10%	4.23%	0.79%	0.26%	7.39%	30.98%
	**Africa (833)**	4.08%	3.96%	6.84%	0	0	0.12%	0.12%	0	7.20%	58.46%
	**Asia (3188)**	11.14%	10.38%	11.70%	0.06%	0.06%	1.60%	1.44%	0.35%	11.79%	34.47%
	**Europe (129)**	14.73%	13.95%	13.95%	0	3.10%	17.83%	1.55%	1.55%	9.30%	65.89%
	**North America (1998)**	5.66%	1.45%	1.55%	0	0	9.26%	0.50%	0.15%	4.35%	24.32%
	**Oceania (1105)**	1.09%	0.81%	0.81%	0	0	1.99%	0.09%	0.09%	1.00%	6.06%
	**South America (438)**	17.12%	15.75%	16.21%	0.46%	0.46%	9.82%	0.23%	0.68%	5.02%	36.30%
**Country**	**Oceania: Australia (691)**	1.74%	1.30%	1.30%	0	0	3.18%	0.14%	0.14%	1.59%	9.70%
	**South America: Brazil (182)**	8.24%	6.59%	7.14%	0.55%	0.55%	2.75%	0	0.55%	4.95%	14.29%
	**North America: Canada (1414)**	5.45%	1.45%	1.77%	0	0	11.24%	0.64%	0.14%	4.95%	25.25%
	**Asia: China (1908)**	5.97%	4.87%	7.02%	0.05%	0.05%	2.62%	2.36%	0.47%	8.86%	29.04%
	**Africa: Nigeria (140)**	0	0	0	0	0	0	0	0	0.71%	62.86%
	**Europe: Switzerland (61)**	8.20%	8.20%	8.20%	0	4.92%	1.64%	3.28%	1.64%	4.92%	75.41%
**Serovar**	**Typhi (3013)**	7.63%	7.60%	7.60%	0.03%	0.03%	0	0.03%	0	8.30%	22.97%
	**Paratyphi A (421)**	0.24%	0.24%	0.24%	0	0	0	0	0	0.24%	0.71%
	**Enteritidis (1424)**	4.00%	3.72%	3.86%	0.07%	0.07%	0.77%	0.14%	0	3.09%	31.32%
	**Typhimurium (589)**	9.34%	6.96%	12.73%	0	0	0.34%	1.02%	0.34%	10.02%	50.93%
	**I 1,4**,[5]**,12:i:- (231)**	13.42%	9.52%	12.99%	0	0	1.73%	3.46%	2.60%	15.58%	58.44%
	**Infantis (219)**	29.22%	28.77%	28.77%	0	0	18.72%	0	0	1.83%	63.47%
	**Heidelberg (162)**	24.69%	1.85%	1.85%	0	0	100.00%	0	0.62%	1.85%	100.00%
	**Virchow (120)**	0	0	1.67%	0	0	1.67%	0	0	1.67%	3.33%
	**Saintpaul (109)**	4.59%	2.75%	2.75%	0	0	0.92%	0	0	0	1.83%
	**Dublin (107)**	25.23%	0	0	0	0	0	0	0	0	38.32%
	**Choleraesuis (92)**	5.43%	5.43%	23.91%	0	0	0	2.17%	4.35%	27.17%	85.87%
	**Panama (84)**	0	0	0	0	0	0	1.19%	1.19%	7.14%	41.67%
	**Newport (64)**	6.25%	3.13%	3.13%	0	0	0	0	0	1.56%	15.63%
	**Javiana (51)**	1.96%	0	1.96%	1.96%	1.96%	0	0	0	0	3.92%
	**Muenchen (51)**	11.76%	11.76%	11.76%	0	0	0	0	0	5.88%	72.55%
**ST**	**ST1 (1835)**	12.43%	12.43%	12.43%	0.05%	0.05%	0	0.05%	0	13.46%	31.72%
	**ST11 (1302)**	4.38%	4.07%	4.22%	0.08%	0.08%	0.84%	0.15%	0	3.38%	33.26%
	**ST2 (1160)**	0.17%	0.09%	0.09%	0	0	0	0	0	0.26%	9.48%
	**ST19 (459)**	5.66%	1.96%	3.27%	0	0	0.44%	1.31%	0.22%	3.70%	28.10%
	**ST85 (252)**	0	0	0	0	0	0	0	0	0	0.79%
	**ST32 (198)**	30.30%	29.80%	29.80%	0	0	19.19%	0	0	1.52%	67.17%
	**ST313 (180)**	18.33%	18.33%	32.22%	0	0	0	0	0	17.78%	88.89%
	**ST15 (161)**	24.22%	1.86%	1.86%	0	0	100.00%	0	0.62%	1.86%	100.00%
	**ST129 (158)**	0.63%	0.63%	0.63%	0	0	0	0	0	0.63%	0.63%
	**ST34 (149)**	16.11%	14.09%	20.81%	0	0	2.01%	4.70%	3.36%	26.17%	91.28%
	**ST16 (115)**	0	0	0	0	0	0	0	0	0	1.74%
	**ST50 (91)**	2.20%	0	0	0	0	0	0	0	0	1.10%
	**ST48 (84)**	0	0	0	0	0	0	1.19%	1.19%	7.14%	41.67%
	**ST10 (80)**	33.75%	0	0	0	0	0	0	0	0	47.50%
	**ST68 (77)**	0	0	22.08%	0	0	0	2.60%	0	27.27%	90.91%

Comparative analysis of clinically critical antibiotic resistance rates in human invasive *Salmonella* infections across different countries, continents, serovars, and STs worldwide. The country with the highest number of isolates per continent is displayed. The top 15 dominant serovars and 15 dominant STs were selected for display.

Among these fluoroquinolone resistance isolates (53.11%), only 7.39% (568) were resistant to ciprofloxacin. The prevalence of ciprofloxacin resistance was highest in Asia (11.79%), especially in China (8.86%), followed by Europe (9.30%) and Africa (7.20%) (Table 1). Hotspots of ciprofloxacin resistance were predicted in *S*. Choleraesuis (ST68), *S*. I 1,4,[5],12:i:- (ST34), *S*. Typhimurium (ST313), and *S*. Typhi (ST1). The prevalence of fosfomycin resistance (4.23%) was the highest in Europe (17.83%), followed by South America (9.82%) and North America (9.26%) (Table 1). 11.24% of 1414 isolates from Canada were fosfomycin resistant. All these *S*. Heidelberg isolates were fosfomycin resistant, followed by *S*. Infantis ST32. Higher prevalence of azithromycin resistance rates (0.79%) was observed in Europe (1.55%, especially in Switzerland (3.28%)) and Asia (1.44%, especially in China (2.36%)) than in other regions (Table 1). Hotspots of azithromycin resistance were predicted in *S*. I 1,4,[5],12:i:- (ST34), *S*. Typhimurium (ST19), *S*. Choleraesuis (ST68), and *S*. Panama (ST48). As for polymyxin resistance (0.26%), the prevalence of colistin resistance was the highest in Europe (1.55%, especially in Switzerland (1.64%)), followed by South America (0.68%) (Table 1). Colistin resistance was frequently identified in *S*. Choleraesuis, *S*. I 1,4,[5],12:i:- (ST34), and *S*. Panama (ST48).

Overall, the resistance levels of clinically critical antibiotics worldwide are markedly serious and are of great concern. In addition, we also observed MDR rates in Europe (65.89%), Africa (58.46%), South America (36.30%), and Asia (34.47%) were above the global average (30.98%) (Table 1). Hotspots of MDR were predicted in Europe, especially in Switzerland (75.41%), and Africa, especially in Nigeria (62.86%). Notably, multiple serovars and STs exhibited high MDR rates.

### Increasing trends of invasive *Salmonella* conferring resistance to clinically critical antibiotics

Generally, we observed that the number of AMR markers per invasive *S*. *enterica* isolate showed a wave-upward trend worldwide, especially in Asia and North America (Supplementary Fig. 10A). These isolates were mainly distributed in *S*. Typhi (ST1 and ST2), *S*. Choleraesuis ST68, *S*. Dublin ST10, *S*. Kentucky ST198, *S*. Typhimurium ST313, and *S*. Paratyphi A ST129 (Supplementary Fig. 10B, C). While decreased trends were mainly identified in *S*. Panama ST48, *S*. Saintpaul ST50, *S*. Typhimurium ST19, *S*. Virchow ST16, and *S*. Infantis ST32.

**Fig. 3:**
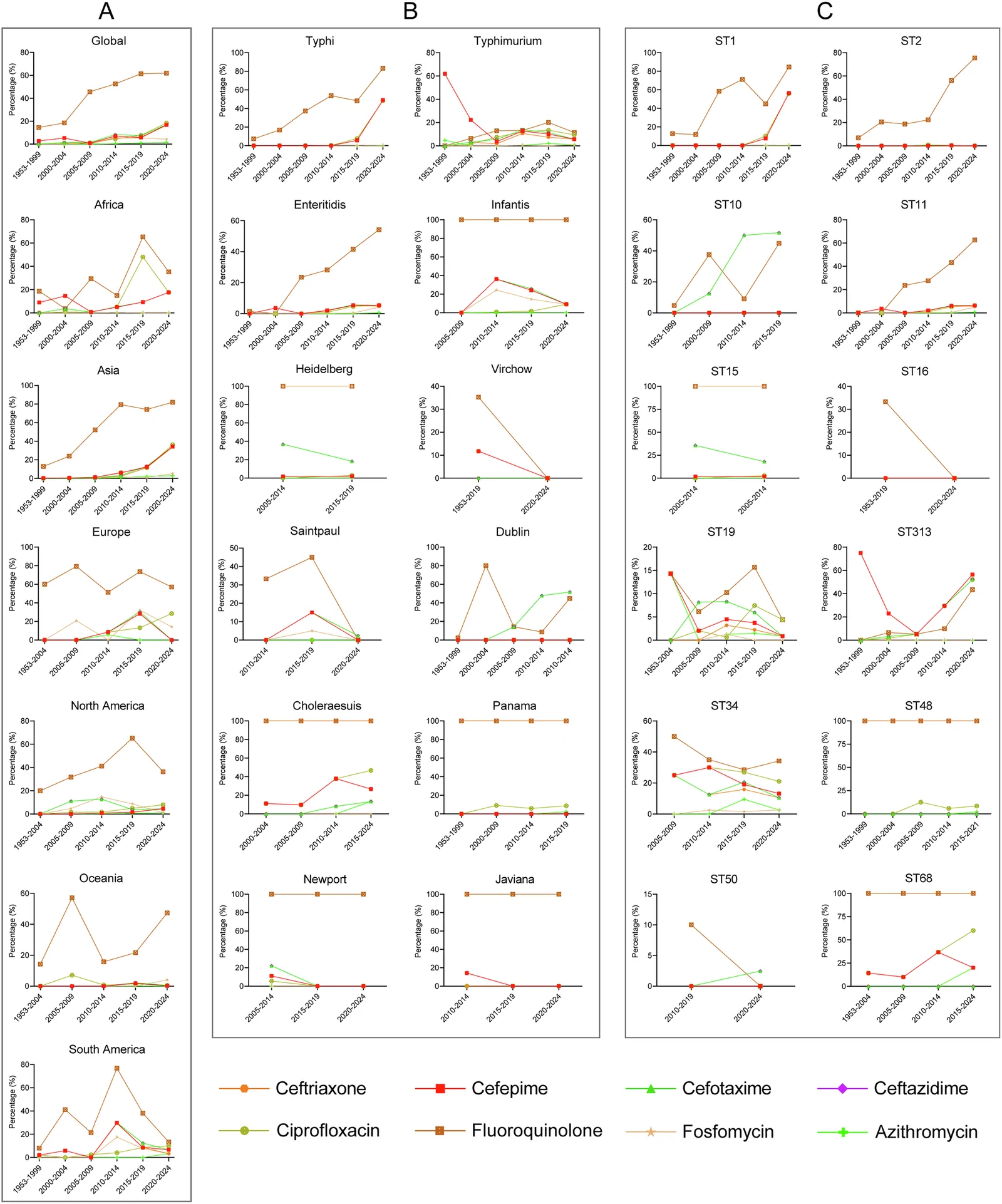
Temporal dynamics of clinically critical antibiotic resistance in *S*. *enterica* causing human invasive infections. **A** Temporal trends of clinically critical antibiotic resistance rates worldwide. **B** Temporal trends of clinically critical antibiotic resistance rates in the top 12 serovars. **C** Temporal trends of antibiotic resistance rates in the top 12 STs. Source data are provided as a Source Data file.

Ciprofloxacin, ceftriaxone, and azithromycin have been recommended as a critical choice for treating invasive *Salmonella* infections. Therefore, we mainly focused on AMR trends in these clinically critical antibiotics. Globally, we observed the proportion of isolates with ciprofloxacin nonsusceptibility within invasive *Salmonella* infections significantly increased from 0.21% before 2000 to 18.42% in the 2020–24 period (Fig. 3A, Supplementary Data 9). The prevalence of increased trends in ciprofloxacin nonsusceptibility was observed in Asia, North America, Europe, and South America (Fig. 3A, Supplementary Data 9). *S*. Typhi, *S*. Enteritidis, *S*. Infantis, and *S*. Choleraesuis made the largest contribution to the increased trend of ciprofloxacin nonsusceptibility (Fig. 3B, Supplementary Data 10), and these collections were mainly distributed in ST1, ST11, ST313, ST32, ST68, and ST198 (Fig. 3C, Supplementary Data 11).

Similar to *bla*_CTX-M_ genes, the proportion of invasive *Salmonella* conferring to 3GCs (for example, ceftriaxone, cefotaxime, and ceftazidime) (from 0.21–17.01%) and 4GC (for example, cefepime) (from 2.87–16.85%) significantly increased over time worldwide (Fig. 3A), including Asia, North America, Europe, and Africa. The proportion of isolates with 3GC and 4GC nonsusceptibility was mainly identified in *S*. Typhi (ST1 and ST2), *S*. Enteritidis ST11, *S*. Dublin ST10, *S*. Kentucky ST198, and *S*. Typhimurium ST313 (Fig. 3B, C).

In the 2024 WHO list of medically important antibiotics, fosfomycin has been listed as the highest priority critically important antibiotic. We also observed that the proportion of isolates with fosfomycin resistance significantly increased over time in Asia (from 0 to 5.18%) and Oceania (from 0 to 4.10%) (Fig. 3A). *S*. Enteritidis ST11 and *S*. Kentucky ST198 were the major contributors in the collection (Fig. 3B, C). In addition, the proportion of isolates with MDR showed a significant increased trend over time in Asia and Africa, especially in *S*. Typhi and *S*. Dublin.

### Co-occurrence patterns of clinically important ARGs and MGEs

**Fig. 4:**
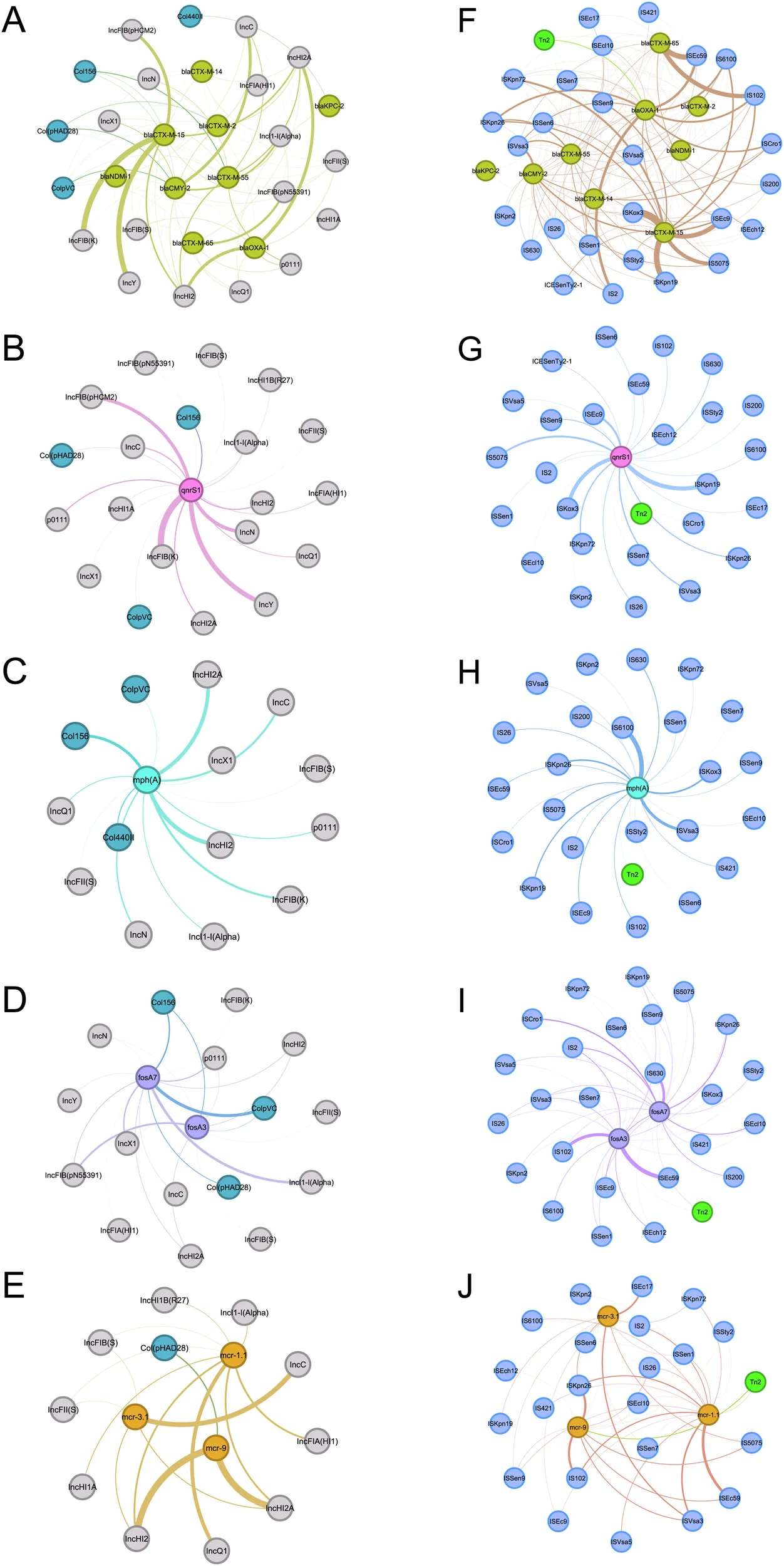
Correlation network between clinically critical ARGs and MGEs. **A** The co-occurrence network between plasmid replicons and 3GCs resistance genes. **B** The co-occurrence network between plasmid replicons and ciprofloxacin and resistance genes. **C** The co-occurrence network between plasmid replicons and azithromycin resistance genes. **D** The co-occurrence network between plasmid replicons and fosfomycin resistance genes. **E** The co-occurrence network between plasmid replicons and polymyxin resistance genes. **F** The co-occurrence network between MGEs and 3GCs resistance genes. **G** The co-occurrence network between MGEs and ciprofloxacin resistance genes. **H** The co-occurrence network between MGEs and azithromycin resistance genes. **I** The co-occurrence network between MGEs and fosfomycin resistance genes. **J** The co-occurrence network between MGEs and polymyxin resistance genes. Source data are provided as a Source Data file.

To investigate the contributors of MGEs to the dissemination of clinically important ARGs, we analyzed and built the co-occurrence networks between representative ARGs and MGEs. For cephalosporin resistance genes, the higher frequency of co-existence between *bla*_CTX-M-15_ and IncFIB(K), IncY, IncFIB(pHCM2), IncHI2, and IncHI2A was observed (Fig. 4A). The higher frequency of co-occurrence values between *bla*_OXA-1_ and IncHI2 and IncHI2A was observed. The fluoroquinolone resistance gene *qnrS1* was mainly identified in IncFIB(K), IncN, and IncY-carrying plasmids (Fig. 4B). The *mph(A)* gene with a higher co-existence frequency and several plasmid replicons, including IncHI2 and IncHI2A (Fig. 4C). The *fosA7* gene was more prevalent in ColpVC-carrying plasmids, while the *fosA3* gene was mainly identified in IncFIB(Pn55391)-carrying plasmids (Fig. 4D). The mobile colistin resistance gene *mcr-1.1*, with a higher frequency of co-existence with IncQ1-carrying plasmids, *mcr-3.1* was mainly identified in IncC-carrying plasmids, while *mcr-9.1* was mainly identified in IncHI2/IncHI2A-carrying plasmids (Fig. 4E).

To better understand the genetic environment of these representative ARGs, we further analyzed the coexistence between ARGs and MGEs. Co-occurrence networks showed that *bla*_CTX-M-15_ had a higher frequency of co-existence with IS*Kox3*, IS*Kpn19*, and IS*Ec9*, while *bla*_CTX-M-65_ had a higher frequency of co-existence with IS*Ec95* and IS*102* (Fig. 4F). The *qnrS1* gene was mainly identified in IS*Kox3* and IS*Kpn19*-carrying contigs (Fig. 4G). The *mph(A)* gene is often located adjacent to IS*6100*, followed by IS*Vsa3* (Fig. 4H). *fosA3* is often located adjacent to IS*Ec59* and IS*102*, *fosA7* is often located adjacent to IS*630* (Fig. 4I). However, different MGEs showed frequent co-existence with *mcr* genes, for example, IS*Ec59* with *mcr-1.1*, IS*Ec19* with *mcr-3.1*, and IS*102* with *mcr-9* (Fig. 4J). Due to the short-read sequencing data we obtained, we could not determine the precise location of the ARGs and MGEs.

### Temporal trends of MGEs and VFs in invasive *Salmonella*

A total of 67 different plasmid replicons were identified (Supplementary Data 12). Some replicons, for example, IncFIB(K), IncFIB(pHCM2), and IncX1 present an obviously upward trend over time (Supplementary Fig. 11A, Supplementary Data 13). IncFIB(S) and IncFII(S) were in a dominant position in Africa, and IncX1 was in a dominant position in Asia, North America, and South America (Supplementary Fig. 11B). In different serovars, IncFIB(S) and IncFII(S) had a dominant proportion in *S*. Enteritidis, *S*. Typhimurium, and *S*. I 1,4,[5],12:i:-. In *S*. Dublin, IncFII(S) and IncX1 had an advantageous proportion (Supplementary Fig. 11C). In different STs, IncFIB(S) and IncFII(S) were in an advantageous position in *S*. Enteritidis ST11, *S*. Typhimurium ST313, and *S*. Typhimurium ST19 (Supplementary Fig. 11D).

A total of 170 determined MGEs were identified (Supplementary Data 14). Globally, IS*Ec9*, IS*Kpn19*, and IS*Sen1* show an upward trend (Supplementary Fig. 12A, Supplementary Data 15). IS*26* and IS*6100* were mainly detected in Europe, IS*Sen6* was mainly detected in Asia, Oceania, and Europe, and IS*Ecl10* and IS*Kpn2* were mainly detected in Africa and Europe (Supplementary Fig. 12B). In *S*. Typhi, IS*Sen6* and IS*Sty2* had an advantage proportion, and in *S*. Paratyphi A, IS*Ech12*, IS*Sen6*, and IS*Sty2* had an advantage proportion. IS*Sen1* and IS*Sen7*, IS*Ecl10* were mainly detected in NTS, for example, *S*. Enteritidis, *S*. Typhimurium, *S*. Saintpaul, and *S*. Dublin (Supplementary Fig. 12C). In different STs, IS*26* was dominantly detected in *S*. Typhimurium ST313 and ST34, and IS*Ech12* was dominantly detected in *S*. Paratyphi A ST85, *S*. Infantis ST32, and *S*. Paratyphi A ST129 (Supplementary Fig. 12D).

A total of 175 different virulence factors (VFs) belonging to 10 different types were identified (Supplementary Data 16). Globally, *lpfA*, *lpfB*, *lpfC*, *lpfD*, and *lpfE*, belonging to adherence, showed an increasing trend (Supplementary Fig. 13A, Supplementary Data 17). *pefA*, *pefB*, *pefC*, and *pefD* mainly existed in Africa, and *tviB*, *tviC*, *tviD*, *tviE*, *vexA*, *vexB*, *tviC*, and *tviD* mainly existed in Asia and Oceania (Supplementary Fig. 13B). *tviB*, *tviC*, *tviD*, *tviE*, *vexA*, *vexB*, *tviC*, and *tviD* mainly observed in *S*. Typhi, and *fyuA*, *irp1*, *irp2*, *ybtA*, *ybtE*, *ybtP*, *ybtQ*, *ybtS*, *ybtT*, *ybtU*, and *ybtX* existed in *S*. Infantis (Supplementary Fig. 13C). *tviB*, *tviC*, *tviD*, *tviE*, *vexA*, *vexB*, *tviC*, and *tviD were* mainly observed in *S*. Typhi ST1 and ST2, and *fyuA*, *irp1*, *irp2*, *ybtA*, *ybtE*, *ybtP*, *ybtQ*, *ybtS*, *ybtT*, *ybtU*, and *ybtX* were mainly observed in *S*. Infantis ST32.

### Phylogenetic relationships of dominant iNTS sequence types, ST34 and ST32

To analyze the population structure characteristics of iNTS, the maximum likelihood tree based on core genome single-nucleotide polymorphisms (SNPs) was established for two representative STs, including ST34 (*S*. Typhimurium and *S*. I 1,4,[5],12:i:-) and *S*. Infantis ST32. Based on 2527 SNPs, global invasive ST34 isolates could be divided into 7 lineages (Supplementary Fig. 14A). L1 consists of strains from Europe (Switzerland) and strain from Asia (China), L2 consists of strains from North America, which are clustered with Canadian strains, L3 consists of strains from Asia, which are clustered with Chinese strains and mostly derived from our sequenced data, L4 mainly consists of strains from Europe (Switzerland), L5 mainly consists of strains from Europe (Switzerland and Germany) and Asia (China), L6 mainly consists of strains from Asia, all of which come from China, and L7 consists of strains from North America (USA and Canada) and Asia (China) (Supplementary Fig. 14A). The variant with phenotype of macro, red, dry, and rough (mrdar)^37^ was identified, and all are distributed in L3, forming an independent clustered lineage (Supplementary Fig. 14A). From the distribution of functional genes, L3 exhibits different characteristics compared to other lineages. The *aac(3)-IV*, *aadA1*, and *aph(4)-Ia*, *ARR-3*, *catB3*, *dfrA12*, and *gyrA* p.D87N genes were mainly observed in L3 (Supplementary Fig. 14A). 3GC’s resistance genes *bla*_CTX-M-14/55_ and *qnrS1* were mainly detected in L3, L6, and L7, and *mph(A)* was detected in L7 (Supplementary Fig. 14A). Adherence-coding gene *shdA* mainly appears clustered in L3.

Based on 2640 SNPs, global invasive ST32 can be divided into 4 clades (Supplementary Fig. 14B). Clade 1 is composed of strains mainly from an African country (South Africa). Clade 2 is mainly composed of North American countries (Canada). Clade 3 is further divided into 3.1 and 3.2, with 3.1 mainly composed of strains from the South American country (Peru) and 3.2 mainly composed of strains from the South American (Peru) and North American countries (Canada and USA) (Supplementary Fig. 14B). The *aadA1*, *aph(3’)-Ia*, *dfrA14*, *sul1*, *tet(A)*, and *gyrA* p.D87Y genes were mainly detected in clade 3, *aac(3)-IV*, *aph(4)-Ia*, *floR*, *fosA3*, and *bla*_CTX-M-65_ were detected in clade 3.2 (Supplementary Fig. 14B). In addition, *floR* and *tet(A)* were detected in clade 1. The distribution of VFs also varies among different clades. Adherence-coding genes *faeD*/*E* and nutritional/metabolic factor-coding genes *fyuA*, *irp1*/*2*, *ybtA*/*X* were mainly detected in clade 3 (Supplementary Fig. 14B).

## Discussion

Cases of iNTS infections have been increasing in China^38^, Australia^39^, the Netherlands^40^, Canada^41^, and elsewhere^42,43^, while the GBD caused by typhoid and paratyphoid fever showed a declining trend^42^. Of greater concern is the increasing prevalence of BSIs and urinary tract infections caused by MDR isolates worldwide. We assembled 1908 invasive isolates in China and combined them with 5783 isolates from 77 countries across 6 continents, covering a period of 70 years, as a comprehensive value resource for further comparative genomics. We discovered two newly identified STs, ST10417 (*S*. Paratyphi A) and ST10419 (*S*. Enteritidis), were isolated from China for the first time. In addition, using genotyping analysis, we identified four genotypes, including 2.3.3, 1, 2.3.2, and 2.4.7, in China; the previous study only identified genotype 2.3.3 from Chinese *S*. Paratyphi A strains^44^. This study describes the genomic epidemiology and AMR evolution of global invasive *Salmonella* by combining newly generated WGS sequences with publicly available data, providing a critical baseline of understanding for this key pathogen, causing both diarrhea and invasive infections (usually BSIs and urinary tract infections) with regional and international genomic-based surveillance. Our findings reveal the presence of substantial geographic, serovar-related, and source-specific variations in clinically critical antibiotic patterns in invasive *Salmonella* isolates.

To the best of our knowledge, this longitudinal genomic epidemiology study spanning 70 years is the largest study of invasive *S*. *enterica* isolates (*n* = 7691) ever performed at the global scale. Globally, we observed an increasing iNTS serovar diversity over time, and an obvious shift in dominant serovars and STs worldwide across 6 continents. A previous study^35^ assembled a WGS dataset (*n* = 1115), and part of it (*n* = 224, 20.1%) was reanalyzed from our previous study^16^, and reported that the majority of invasive infection cases occurred in southeastern coastal regions, with fewer cases reported in northern areas. A recent study^45^ reported that *S*. *enterica* was listed in one of the ten bloodstream infection bacterial pathogens in China, and the detection rate was higher in Southern China than in Eastern, Northern, or Central China. Our data showed invasive infections were dominantly distributed in Guangxi (*n* = 815), followed by Fujian (*n* = 211), and Shanghai (*n* = 172).

Despite regional and WHO surveillance efforts, gaps remain in understanding spatial disparities and temporal trends of first- and second-line antibiotics (including ciprofloxacin, 3GC, 4GC, and azithromycin) at the global scale^43^. In this study, we provide a global genomic surveillance report for invasive *S*. *enterica* from 77 countries using the largest invasive genomic dataset (*n* = 7691). Our data showed that the majority (74.7%) of invasive *S*. *enterica* isolates in this study were susceptible to beta-lactams, and the resistant rate (25.3%) was higher than that in a previous study (18.3%) at a global scale^22^. Our colleagues previously reported that 3.9% (7/178) of iNTS infections showed co-resistance to 3GCs and ciprofloxacin, and a steady increase in the proportion of cefotaxime- and ciprofloxacin-resistant isolates in China during 2007–2016^38^. We observed increasing trends in clinically critical antibiotics, including ESBLs, including 3GCs (ceftriaxone, cefotaxime, and ceftazidime), 4GC (cefepime), ciprofloxacin, and azithromycin worldwide during 1953–2024, except for Oceania and South America, which have emerged as a critical public health and biosafety challenge. These findings are in accordance with many other regional, national, or international studies^10,11,24,43,46–51^. Of note, our data show the emergence of iNTS (for example, *S*. Enteritidis ST11, *S*. Dublin ST10, *S*. Kentucky ST198, *S*. Typhimurium ST313, and *S*. Infantis ST32) as the predominant contributor to the prevalence of ESBLs, ciprofloxacin, and azithromycin resistance in *S*. *enterica* causing bloodstream infections worldwide. Although the increasing trends were different in different serovars and STs across different countries, the scenario in dominant clones causing BSIs and urinary tract infections worldwide is of great concern.

Our study has several limitations. First, although we used the comprehensive passive surveillance and the best publicly available data, differences remained in the number of genomes from different countries, serovars, STs, and sampling years, which may indicate a potential sampling bias. Second, even though 585,220 genomes were downloaded from NCBI (as of February 14, 2025), 117,461 with comprehensive metadata are of human origin, and only 6781 were recorded as invasive infections. Third, a total of 148 serovars and 315 STs were identified, and only twenty predominant clones were selected for AMR analysis. Fourth, antibiotic consumption habits (records of clinical treatments) and antibiotic usage may influence AMR, those was not taken into account. Fifth, AMR phenotypes were predicted using WGS prediction. And the analysis of AMR mechanisms is currently limited to acquired genes and chromosomal mutations recorded in the ResFinder and PointFinder databases^52,53^. For example, for azithromycin resistance, a comprehensive investigation including all chromosomal mutations (for example, the ribosome target site (e.g., 23S rRNA) and ribosomal proteins L4 and L22) is needed. The previous study reported that *acrB* mutations are the main driver of azithromycin resistance in typhoidal *Salmonella* in Bangladesh, and it was also observed increased trend worldwide^54^. Last, although co-existence between some representative ARGs and MGEs was revealed, the precise context of mobile ARGs and MGEs was not investigated; a long-read sequencing approach needs to be used in the future. Timely global WGS surveillance on AMR and data sharing are crucial to refine national and international invasive *Salmonella* treatment guidelines and AMR control^26,27,55^.

In summary, our study confirms the dominant serovar and ST population switch in the extra-intestinal invasive pathogenic *S*. *enterica* worldwide, which is under adaptive selection. This large worldwide longitudinal genomic collection allowed us to identify the emergence of AMR in dominant *Salmonella* serovars/STs and determine their contribution to rising BSIs and urinary tract infections, as well as AMR levels. We observed a relatively high level of clinically critically important antibiotics, including ciprofloxacin (7.39%), 3GCs (7.91%), 4GC (7.27%), fosfomycin (4.23%), and azithromycin (0.79%), in blood-invasive *S*. *enterica* isolates, and obvious geographic, serovar-related, and source-specific variations also existed. The increasing prevalence of *Salmonella* isolates from BSIs and urinary tract infections, conferring to ciprofloxacin, 3GC, 4GC, and azithromycin, is posing a huge public health challenge. This highlights the value of integrating genomic surveillance for decision-makers in formulating effective AMR control and prevention of the dissemination of high-risk MDR and extensively drug-resistant clones. We have a comprehensive microbial strain database and an open-access genomic data platform (https://nmdc.cn/clsgdbv2/), and we welcome collaborations with researchers worldwide.

## Methods

### Ethical Statement

All *Salmonella* strains were collected under the support of the China-US emerging infectious diseases cooperation project (No. 1U2GGH00961-01). All isolates were collected under informed consent or approval in the local sentinel hospitals or public laboratories. No clinical data were collected as part of this study.

### Study design and data collection

In this retrospective genomic epidemiological study, we analyzed *S*. *enterica* isolates causing human invasive infections (sourced from the CLSSSDB) and a public database on the National Center of Biotechnology Information (NCBI, https://www.ncbi.nlm.nih.gov/) to investigate the global population structure and AMR trends of *S*. *enterica*. We included 621 isolates from six Chinese PLAs (new generated WGS data), including Shanghai, Guangxi, Fujian, Shanxi, Xinjiang, and Chongqing in China from January 1, 1994, to December 31, 2023, and 289 isolates from the CLSGDBv2 (https://nmdc.cn/clsgdbv2) was reported in our previous studies^16,21^, as well as 6781 strains with comprehensive metadata and manually screening recorded in the NCBI (As of Feb 14, 2025)^22^. A total of 585,220 publicly available *Salmonella* genomes and BioSample information were downloaded and collected. After screening by *Salmonella* Serotyping by Whole Genome Sequencing (SeqSero2 version 1.3.1)^56^, *Salmonella* In Silicon Typing Resource (SISTR version 1.1.2)^57^, and multilocus sequence typing (MLST version 2.22.0)^58^ with default parameters based on the seven housekeeping genes: *dnaN*, *aroC*, *hisD*, *hemD*, *sucA*, *purE*, and *thrA*, a total of 6781 human invasive *S*. *enterica* strains were used for downstream analysis. The serovars for each isolate (*n* = 844) recorded in the CLSSSDB were confirmed using the Kauffmann-White Scheme with O- and H-antigen specific sera.

### DNA extraction and WGS

Isolates recorded in the CLSSSDB were whole-genome sequenced using the Illumina NovaSeq 6000 platform as part of the Chinese Local *Salmonella* Genome DataBase^16,21^. Detailed information about DNA extraction, WGS, quality control, assembly, and annotation is provided as previously described^16^. Briefly, fastp version 0.23.4^59^ was used to remove low-quality sequences and residual adapter sequences. High-quality reads were assembled using Unicycler version 0.4.8^60^. Protein-coding sequences (CDSs) were annotated using Prokka version 1.14.5^61^.

### Bioinformatics analysis

WGS data without STs were submitted to the Enterobase (https://enterobase.warwick.ac.uk/) to assign new STs. The thresholds of functional genetic determinants are consistent with previous studies^16,21,22^. ResFinder version 4.1^52,53^ (coverage ≥ 90% and identity ≥ 95%) was used to identify acquired AMR genes and chromosomal gene mutations mediating AMR. The resistance phenotypes of the isolates were predicted based on AMR genotypes. PlasmidFinder version 2.1^62^ (coverage ≥ 90% and identity ≥ 95%) was used to identify plasmid replicons. MobileElementFinder version 1.0.5^63^ (coverage ≥ 90% and identity ≥ 90%) was used to identify MGEs. SPIFinder version 2.0^64^ (coverage ≥ 60% and identity ≥ 95%) was used to identify *Salmonella* pathogenic islands (SPIs). ABRicate version 1.0.1 was used to identify VFs with default parameters (coverage ≥ 80% and identity ≥ 80%)^21^. The genotypes for *S*. Paratyphi A were determined using Paratype version 1.1^44^ with the following parameters: --read_cutoff 0.75, --phrd_cutoff 20, and --mapq_cutoff 20. The genotypes for *S*. Typhi were determined using GenoTyphi version 2.10^65^ with default parameters (--species typhi).

To investigate the evolutionary dynamics of the invasive *S*. *enterica* population, we selected three representative clones, including ST34 and ST32. High-quality reads were mapped against reference genomes (*S*. Typhimurium str. LT2: accession no. AE006468, Infantis ST32: accession no. GCA_001931595.1) to identify core genome single-nucleotide polymorphisms (SNPs) using Snippy version 4.7.0 (mincov = 10, minfrac = 0.9, mapqual = 60, basequal = 13, minqual = 100, and ram = 8). Gubbins version 3.4^66^ was used to remove recombinant regions and invariant sites. SNP-sites version 2.5.1^67^ was used to extract the final SNPs. For the ST34 isolates, a total of 2535 SNPs were detected in the core genome alignment before recombination removal, and 2527 SNPs remained after filtering recombinant regions. For the ST32 isolates, 3039 SNPs were identified before recombination removal, and 2640 SNPs were retained after recombination filtering. RAxML version 8.2.12^68^ was used to build a maximum likelihood estimation phylogenetic tree. To account for rate variation across sites, a Gamma-distributed rate heterogeneity model was integrated with the General Time Reversible (GTR) nucleotide substitution model for the analysis. Nodal support was evaluated through a combined rapid bootstrap and maximum likelihood search, utilizing 1000 bootstrap replicates. To ensure reproducible results, the random seeds for generating the initial parsimony tree and for the bootstrap resampling procedure were both fixed at 12345.

### Statistical analysis and data visualization

Multiple comparisons among different groups (with the Kruskal–Wallis test and Mann–Whitney U tests) were performed using GraphPad Prism version 9.5.0. Statistical comparisons were conducted using a non-parametric one-way ANOVA for analyses involving multiple groups, and a *t*-test for direct comparisons between two groups. Stack columns, histograms, and pie charts were built using GraphPad Prism version 9.5.0. Global sampling locations of Salmonella genomes were mapped in R (v4.5.2) using the ggplot2 and sf packages. Medium-scale country boundary data were obtained from the Natural Earth database. The map was generated with the Robinson projection, with countries colored by continent. GrapeTree v1.5.0^69^ was used for MST analysis and visualization. Heatmaps were generated on the ImageGP platform (http://www.ehbio.com/ImageGP/). The co-occurrence network graph of ARGs and plasmid replicons was generated using Gephi v0.10.1^70^. The iTOL v7.0^71^ was used to visualize and annotate phylogenetic trees.

### Reporting summary

Further information on research design is available in the Nature Portfolio Reporting Summary linked to this article.

## Supplementary information

Supplementary_Information

Description of Additional Supplementary Files

Supplementary Dataset 1–17

Reporting Summary

Transparent Peer Review file

## Source data

source data

## Data Availability

The WGS data generated in this study have been deposited in the NCBI database under accession PRJNA1313186. The publicly available genomes were downloaded from the NCBI database, and accession numbers are available in Supplementary Data 1, 2. Source data are provided with this paper.

## Code availability

The source code used for invasive *Salmonella* analysis in this study is available at https://github.com/NANYW123/Global-invasive-Salmonella.git. All codes and tools used in this study were available from the following public repository: Fastp version 0.23.4 (https://github.com/OpenGene/fastp), Unicycler version 0.4.8 (https://github.com/rrwick/Unicycler), Prokka version 1.14.5 (https://github.com/tseemann/prokka), SeqSero2 version 1.3.1 (https://github.com/denglab/SeqSero2), SISTR version 1.1.2 (https://github.com/phac-nml/sistr_cmd), MLST version 2.22.0, https://github.com/tseemann/mlst), Enterobase (http://enterobase.warwick.ac.uk), ResFinder version 4.1 (https://bitbucket.org/genomicepidemiology/resfinder/src/master), ABRicate version 1.0.1 (https://github.com/tseemann/abricate), PlasmidFinder version 2.1 (https://bitbucket.org/genomicepidemiology/plasmidfinder), MobileElementFinder version 1.0.5 (https://cge.cbs.dtu.dk/services/MobileElementFinder), SPIFinder version 2.0 (https://bitbucket.org/genomicepidemiology/spifinder/src/master), GenoTyphi version 2.1 (https://github.com/typhoidgenomics/genotyphi), Paratype version 1.1 (https://github.com/CHRF-Genomics/Paratype), Snippy version 4.7.0 (https://github.com/tseemann/snippy), Gubbins version 3.4 (https://github.com/nickjcroucher/gubbins), SNP-sites version 2.5.1 (https://github.com/sanger-pathogens/snp-sites), snp-dists version 0.8.2 (https://github.com/tseemann/snp-dists), RAxML version 8.2.12 (https://github.com/stamatak/standard-RAxML), GrapeTree version 1.5.0 (https://github.com/achtman-lab/GrapeTree), and Gephi version 0.10.1 (https://github.com/gephi/gephi). Source data are provided with this paper.
