# Author Correction: Global genomic surveillance uncovers emergence and spatiotemporal patterns of World Health Organization priority antibiotic resistance in invasive *Salmonella*

**DOI:** 10.1038/s41467-026-79202-4

**Published:** 2026-10-30

**Authors:** Yuhang Pei, Ziru Yang, Xiaolu Pang, Wufuer. Xiayidan, Meilian Huang, Jinhua Meng, Yufeng Qiu, Mingliu Wang, Hongxia Yang, Chunmei Jing, Mei Zeng, Yue Liu, Wenqing Wang, Yibin Zhou, Sufang Ma, Na Lyu, Mengqi Qu, Chaochao Zhao, Jiangtao Wang, Jiale Chen, Baoli Zhu, George F. Gao, Xuebin Xu, Yanan Wang

**Affiliations:** 1 International Joint Research Center of National Animal Immunology, College of Veterinary Medicine, Henan Agricultural University, Zhengzhou, China; 2 Longhu Laboratory of Advanced Immunology, Zhengzhou, China; 3 Department of Clinical Laboratory, The Fourth People’s Hospital of Nanning, Nanning, China; 4 Department of Microbiology, Xinjiang Uygur Autonomous Region Center for Disease Control and Prevention, Urumqi, China; 5 Department of Laboratory Medicine, Children’s Hospital of Fudan University (Xiamen Branch), Xiamen Children’s Hospital, Xiamen, China; 6 Center of Clinical Medical Laboratory, Shanxi Children’s Hospital, Taiyuan, China; 7 Fujian Center for Disease Control and Prevention, Fujian Provincial Key Laboratory of Zoonosis Disease Research, Fuzhou, China; 8 Guangxi Zhuang Autonomous Region Center for Disease Control and Prevention, Nanning, China; 9 Shanxi Center for Disease Control and Prevention, Taiyuan, China; 10 Children’s Hospital of Chongqing Medical University, Chongqing, China; 11 Department of Infectious Diseases, Children’s Hospital of Fudan University, Shanghai, China; 12 Division of Pathogen Testing and Analysis, Shanghai Municipal Center for Disease Control and Prevention, Shanghai, China; 13 Department of Microbiology, Shanghai Pudong New Area Center for Disease Control and Prevention (Shanghai Pudong New Area Health Supervision Institute), Shanghai, China; 14 Department of Infectious Disease Control, Center for Disease Control and Prevention of Minhang District, Shanghai, China; 15 Beijing Key Laboratory of Antimicrobial-Resistant Pathogen Microbiology and AI-Empowered Containment, Institute of Microbiology, Chinese Academy of Sciences, Beijing, China; 16 University of Chinese Academy of Sciences, Beijing, China; 17 Chinese Center for Disease Control and Prevention (China CDC), Beijing, China

**Keywords:** Bacterial infection, Data mining, Policy and public health in microbiology, Clinical microbiology

Correction to: *Nature Communications* https://doi.org/10.1038/s41467-026-76354-1, published online 18 August 2026

In the version of this article initially published, in the first sentence of the Discussion, a citation to the “World Health Organization. Global antibiotic resistance surveillance report 2025: WHO Global Antimicrobial Resistance and Use Surveillance System (GLASS). World Health Organization (2025)” was missing and is now cited as ref. 43. Subsequent references have been renumbered. The changes are made in the HTML and PDF versions of the article.

