# Supplementary material for: Global genomic surveillance uncovers emergence and spatiotemporal patterns of World Health Organization priority antibiotic resistance in invasive Salmonella
Source: Nat Commun. 2026 Aug 18;17:9871. doi: 10.1038/s41467-026-76354-1 (PMC13578455; doi:10.1038/s41467-026-76354-1)
Supplement: Supplementary file 1 [file 41467_2026_76354_MOESM2_ESM.pdf]

Title: Supplementary Data 1.
Description: Metadata of invasive *Salmonella* isolates from China's national passive surveillance database.

Title: Supplementary Data 2.
Description: Background of public genomic datasets for invasive *Salmonella* used in this study.

Title: Supplementary Data 3.
Description: Global frequency of ARGs in invasive *Salmonella*.

Title: Supplementary Data 4.
Description: Global temporal trends of ARGs in invasive *Salmonella* worldwide.

Title: Supplementary Data 5.
Description: Temporal trends of ARGs among the top 16 invasive *Salmonella* serovars.

Title: Supplementary Data 6.
Description: Temporal trends of ARGs among the top 16 invasive *Salmonella* sequence types (STs).

Title: Supplementary Data 7.
Description: Temporal trends of ARGs in invasive *Salmonella* by continent and top 10 serovars.

Title: Supplementary Data 8.
Description: Distribution of specific antimicrobials in invasive *Salmonella* by continent, major countries, top 15 serovars, and top 15 STs.

Title: Supplementary Data 9.
Description: Continental temporal trends of specific antimicrobials in invasive *Salmonella*.

Title: Supplementary Data 10.
Description: Temporal trends of specific antimicrobials among the top 16 invasive *Salmonella* serovars.

Title: Supplementary Data 11.
Description: Temporal trends of specific antimicrobials among the top 16 invasive *Salmonella* STs.

Title: Supplementary Data 12.
Description: Global frequency of plasmid replicons in invasive *Salmonella*.

Title: Supplementary Data 13.
Description: Global and continental temporal prevalence of plasmid replicons in invasive *Salmonella*.

Title: Supplementary Data 14.
Description: Global frequency of mobile genetic elements (MGEs) in invasive *Salmonella*.

Title: Supplementary Data 15.
Description: Global and continental temporal trends of MGEs in invasive *Salmonella*.

Title: Supplementary Data 16.
Description: Frequency of virulence factors (VFs) among global invasive *Salmonella* isolates.

Title: Supplementary Data 17.
Description: Temporal trends of virulence factors (VFs) in invasive *Salmonella* across global periods.
