# Supplementary material for: Global genomic surveillance uncovers emergence and spatiotemporal patterns of World Health Organization priority antibiotic resistance in invasive Salmonella
Source: Nat Commun. 2026 Aug 18;17:9871. doi: 10.1038/s41467-026-76354-1 (PMC13578455; doi:10.1038/s41467-026-76354-1)
Supplement: Supplementary file 2 [file 41467_2026_76354_MOESM4_ESM.pdf]

nature portfolio

Corresponding author(s): Yanan Wang

Last updated by author(s): Jul 1, 2026

Reporting Summary

Nature Portfolio wishes to improve the reproducibility of the work that we publish. This form provides structure for consistency and transparency in reporting. For further information on Nature Portfolio policies, see our Editorial Policies and the Editorial Policy Checklist.

Statistics

For all statistical analyses, confirm that the following items are present in the figure legend, table legend, main text, or Methods section.

n/a	Confirmed	
☐	☒	The exact sample size (*n*) for each experimental group/condition, given as a discrete number and unit of measurement
☐	☒	A statement on whether measurements were taken from distinct samples or whether the same sample was measured repeatedly
☐	☒	The statistical test(s) used AND whether they are one- or two-sided<br>*Only common tests should be described solely by name; describe more complex techniques in the Methods section.*
☐	☒	A description of all covariates tested
☐	☒	A description of any assumptions or corrections, such as tests of normality and adjustment for multiple comparisons
☐	☒	A full description of the statistical parameters including central tendency (e.g. means) or other basic estimates (e.g. regression coefficient) AND variation (e.g. standard deviation) or associated estimates of uncertainty (e.g. confidence intervals)
☐	☒	For null hypothesis testing, the test statistic (e.g. *F*, *t*, *r*) with confidence intervals, effect sizes, degrees of freedom and *P* value noted<br>*Give P values as exact values whenever suitable.*
☒	☐	For Bayesian analysis, information on the choice of priors and Markov chain Monte Carlo settings
☒	☐	For hierarchical and complex designs, identification of the appropriate level for tests and full reporting of outcomes
☒	☐	Estimates of effect sizes (e.g. Cohen's *d*, Pearson's *r*), indicating how they were calculated

Our web collection on statistics for biologists contains articles on many of the points above.

Software and code

Policy information about availability of computer code

Data collection	wget 1.21.4, fastp v0.23.4, Unicycler v0.4.8, SISTR v1.1.2, SeqSero2 v1.3.1, MLST v2.22.0, ResFinder 4.1, PlasmidFinder v2.1, PointFinder, ABRicate v1.0.1, Prokka v1.14.5, MobileElementFinder v1.0.5, SPIFinder version 2.0, GenoTyphi v2.1, Paratype v1.1, Snippy v4.7.0, Gubbins v3.4, SNP-sites v2.5.1, snp-dists v0.8.2, RAxML v8.2.12, Gephi v0.10.1, etc. Full details are in the Materials and Methods section.
Data analysis	GraphPad Prism v9.0, GrapeTree v1.5.0, excel 2021, R v4.5.2, Snippy v4.7.0, iTOL v7.0, ImageGP platform, etc. Full details are in the Materials and Methods section.

For manuscripts utilizing custom algorithms or software that are central to the research but not yet described in published literature, software must be made available to editors and reviewers. We strongly encourage code deposition in a community repository (e.g. GitHub). See the Nature Portfolio guidelines for submitting code & software for further information.

Data

Policy information about availability of data

All manuscripts must include a data availability statement. This statement should provide the following information, where applicable:

- Accession codes, unique identifiers, or web links for publicly available datasets
- A description of any restrictions on data availability
- For clinical datasets or third party data, please ensure that the statement adheres to our policy

First, a total of 585,220 publicly available Salmonella genomes were downloaded from the National Center for Biotechnology Information (as of February 14, 2025),

after screening by using SeqSero2, STSTR, and MLST, as well as clear metadata information. A total of 260,195 high-quality genomes were used for the following analysis. Among them, 117,461 strains were isolated from humans, 6,781 strains were isolated from blood, urine, and others from extra-intestinal infection sites, and were further selected for AMR analysis. Second, 289 genomes were included from CLSGDBv2 (https://nmdc.cn/clsgdbv2) that were previously published in our previous study. In addition, a total of 621 new generated WGS data in this study from six Chinese provincial-level administrative regions from January 1, 1994, to December 31, 2023. The newly generated WGS data were deposited in the NCBI database under BioProject PRJNA1313186.

Research involving human participants, their data, or biological material

Policy information about studies with human participants or human data. See also policy information about sex, gender (identity/presentation), and sexual orientation and race, ethnicity and racism.

Reporting on sex and gender	In this study, gender was determined based on self-reporting. Participants of both sexes were include.
Reporting on race, ethnicity, or other socially relevant groupings	This study did not use or report any socially constructed or socially relevant categorization variables (such as race, ethnicity, nationality, or socioeconomic status). The research is a retrospective genomic analysis of Salmonella bacterial isolates. Data were limited to bacterial genomic sequences and associated anonymized isolate metadata (e.g., collection date, geographic location, serovar). No individual-level demographic, clinical, or social data were collected or analyzed. Therefore, definitions, classification methods, and control for confounding of such variables are not applicable to this work.
Population characteristics	This study analyzed 7,691 Salmonella genomic data, including 6,781 publicly available sequences, 289 genomes from our Chinese local Salmonella genome database version 2 (https://nmdc.cn/clsgdbv2) and 621 newly sequenced isolates from 621 individuals. Detailed individual-level demographic or clinical data were not collected.
Recruitment	The 621 newly sequenced isolates were obtained from passive surveillance systems in six Chinese provincial-level administrative areas.
Ethics oversight	All isolates were collected with informed consent or local institutional approvals at participating sentinel hospitals or public health laboratories under the China-US emerging infectious diseases cooperation project. This study involved no clinical data collection and utilized anonymized bacterial genomic data.

Note that full information on the approval of the study protocol must also be provided in the manuscript.

Field-specific reporting

Please select the one below that is the best fit for your research. If you are not sure, read the appropriate sections before making your selection.

[x] Life sciences [ ] Behavioural & social sciences [ ] Ecological, evolutionary & environmental sciences

For a reference copy of the document with all sections, see nature.com/documents/nr-reporting-summary-flat.pdf

Life sciences study design

All studies must disclose on these points even when the disclosure is negative.

Sample size	A total of 585,220 publicly available Salmonella genomes were retrieved from NCBI (as of February 14, 2025). After quality control and filtering using bioinformatic tools (including STSTR, SeqSero2, and MLST) and metadata validation, 6,781 genomes were retained. These were combined with 621 newly sequenced isolates from this study and 289 genomes from our Chinese local Salmonella genome database version 2 (https://nmdc.cn/clsgdbv2), resulting in a final dataset of 7,691 genomes used for downstream analysis. Within this collection, a total of 148 serovars and 315 sequence types (STs) were identified.
Data exclusions	First, we screened and summarized the metadata of publicly available Salmonella genomes from the NCBI BioSample (as of February 14, 2025), and isolates with information on the collection time, isolation source, and sampling sites are used for next step analysis. A total of 314,033 genomes were discarded because no background information on the collection time, isolation source, and sampling sites was simultaneously recorded. Second, in silico serotyping was carried out by both the Salmonella in silico typing resource (SISTR v1.1.2) and Salmonella Serotyping by whole genome sequencing (SeqSero2 v1.3.1). Only the prediction result matching between the SeqSero2 and SISTR is used for downstream analysis. Third, multilocus sequence typing (MLST v2.0) was used to assign sequence types (STs) to these genomes based on seven housekeeping genes for examining bacterial relatedness. A total of 10,991 genomes were discarded because no serotype or sequence typing information was predicted. A total of 260,195 high-quality genomes were used for the following analysis. Among them, 117,461 strains were isolated from humans, 6,781 strains were isolated from blood, urine, and others from extra-intestinal infection sites, and were further selected for AMR analysis.
Replication	In this work, we used the established analysis pipeline which has been used to analyze partial data of the genome (https://github.com/NANYW123/Global-invasive-Salmonella.git).
Randomization	This work did not include any interventions and thus the randomization (as used in clinical trials or intervention studies) was not appropriate.
Blinding	This work did not include any interventions and thus the conventional blinding (as used in clinical trials or intervention studies was not appropriate. To obtain a high-quality Salmonella genome dataset, background information for each isolate, sampling time, isolation source (host), geographic location (country and continent), serovar, and ST were screened.

Reporting for specific materials, systems and methods

We require information from authors about some types of materials, experimental systems and methods used in many studies. Here, indicate whether each material, system or method listed is relevant to your study. If you are not sure if a list item applies to your research, read the appropriate section before selecting a response.

Materials & experimental systems

n/a	Involved in the study
☒	☐ Antibodies
☒	☐ Eukaryotic cell lines
☒	☐ Palaeontology and archaeology
☒	☐ Animals and other organisms
☒	☐ Clinical data
☒	☐ Dual use research of concern
☒	☐ Plants

Methods

n/a	Involved in the study
☒	☐ ChIP-seq
☒	☐ Flow cytometry
☒	☐ MRI-based neuroimaging

Plants

Seed stocks	*Report on the source of all seed stocks or other plant material used. If applicable, state the seed stock centre and catalogue number. If plant specimens were collected from the field, describe the collection location, date and sampling procedures.*
Novel plant genotypes	*Describe the methods by which all novel plant genotypes were produced. This includes those generated by transgenic approaches, gene editing, chemical/radiation-based mutagenesis and hybridization. For transgenic lines, describe the transformation method, the number of independent lines analyzed and the generation upon which experiments were performed. For gene-edited lines, describe the editor used, the endogenous sequence targeted for editing, the targeting guide RNA sequence (if applicable) and how the editor was applied.*
Authentication	*Describe any authentication procedures for each seed stock used or novel genotype generated. Describe any experiments used to assess the effect of a mutation and, where applicable, how potential secondary effects (e.g. second site T-DNA insertions, mosiacism, off-target gene editing) were examined.*
