# Supplementary material for: Global genomic surveillance uncovers emergence and spatiotemporal patterns of World Health Organization priority antibiotic resistance in invasive Salmonella
Source: Nat Commun. 2026 Aug 18;17:9871. doi: 10.1038/s41467-026-76354-1 (PMC13578455; doi:10.1038/s41467-026-76354-1)
Supplement: Supplementary file 3 [file 41467_2026_76354_MOESM5_ESM.pdf]

nature portfolio

# Peer Review File

# Global genomic surveillance uncovers emergence and spatiotemporal patterns of World Health Organization priority antibiotic resistance in invasive Salmonella

Corresponding Author: Professor Yanan Wang

**This file contains all reviewer reports in order by version, followed by all author rebuttals in order by version.**

Version 0:

Reviewer comments:

Reviewer #1

(Remarks to the Author)
The author analysed 7691 invasive Salmonella isolates from 77 countries across 6 continents, covering a period of 70 years. This longitudinal genomic epidemiology study is the largest invasive S. enterica isolates ever performed at a global scale.

They observed an increasing iNTS serovar diversity over time, and an obvious shift in dominant serovars and STs worldwide across 6 continents. The majority of invasive S. enterica isolates in this study were susceptible to beta-lactams, and the resistant rate was higher than that in a previous study at a global scale.

They also observed increasing trends in clinically critical antibiotics, including ESBLs, including 3GCs (ceftriaxone, cefotaxime, and ceftazidime), 4GC (cefepime), ciprofloxacin, and azithromycin worldwide during 1953-2024, except for Oceania and South America.

This study highlights the value of integrating genomic surveillance for decision-makers in formulating effective AMR control and prevention of the dissemination of high-risk MDR and extensively drug-resistant clones.

Minor comments;

1) Line 553-554, “ResFinder version 4.1 (coverage ≥ 90% and identity ≥ 95%), PlasmidFinder version 2.1 (coverage ≥ 60% and identity ≥ 90%),”

What were the reason behind choosing the coverage of 90% for ResFinder and coverage of 60% for PlasmidFinder ?

In principle, ResFinder uses sequence alignment in gene level. Meanwhile, PlasmidFider uses sequence alignment in replicon level which is around 100-300 bps.
I think for Plasmid Finder the coverage should be high (more than 90%) as you target small sequence of replicon.

2). The authors used both ResFinder and ABRicate for AMR gene determination. Why did you use both AMR tools ? Which results did you use ResFinder and which ones you used ABRicate and why ?

3). The authors used Gubbins to remove recombination sites.
It would be beneficial for readers to specify the number of total prior and after recombination removal.

Reviewer #2

(Remarks to the Author)
This manuscript describes a large population genomic study (7,691 isolates) of Salmonella enterica susbsp. enterica to study prevalence and spatiotemporal trends of antimicrobial resistance within this subspecies. The spatiotemporal aspect of this study makes this an interesting topic of importance to a wide audience. There are several aspects of the manuscript that

have to be addressed that would improve the readability and correctness.
Throughout the article the authors suggest they are presenting data on antibiotic resistance, susceptibility and non-susceptibility; this suggest a understanding of the resistance phenotype of the isolates; this is not the case, everything in this article is based on genomic data. The manuscript should be revised to make it abundantly clear that it is not the ‘prevalence and spatiotemporal trends of antimicrobial resistance’ that is presented, but the prevalence and spatiotemporal trends of antimicrobial resistance conferring genes and mutations.
Given the format/flow of the article (introduction, results, discussion, materials and methods), it would be good to introduce some of the methodology in the results section to give the reader the information to understand the results presented. For example, what were the inclusion criteria for genomes? Given the fact that data for 548,830 genomes was available to the authors, this may explain to the reader why only a small fraction was used and that a lot of data from human cases of the USA is not included. Include which genotyping methods, MLST or wgMLST schemes were used.
When STs are mentioned, always include the serovar name associated with the ST (if possible) to increase readability.

The title does not seem to reflect the subject of the manuscript; it suggests the emergence of a (single) ciprofloxacin and ceftriaxone-resistant clone. Please revise the title.

**Open Access** This Peer Review File is licensed under a Creative Commons Attribution 4.0 International License, which permits use, sharing, adaptation, distribution and reproduction in any medium or format, as long as you give appropriate credit to the original author(s) and the source, provide a link to the Creative Commons license, and indicate if changes were made.
In cases where reviewers are anonymous, credit should be given to 'Anonymous Referee' and the source.
The images or other third party material in this Peer Review File are included in the article’s Creative Commons license, unless indicated otherwise in a credit line to the material. If material is not included in the article’s Creative Commons license and your intended use is not permitted by statutory regulation or exceeds the permitted use, you will need to obtain permission directly from the copyright holder.
To view a copy of this license, visit https://creativecommons.org/licenses/by/4.0/

**Point-by-point response to the Reviewers**

We thank the Reviewers for their valuable feedback. We have revised our manuscript and addressed the comments raised by the reviewers and editor, and we believe that the manuscript is now substantially improved. Below is a point-by-point response to the Reviewers' comments in blue, and a revised manuscript with track changes highlighted is also attached. In addition to the points mentioned above, we added a new "Code availability" to the Materials and Methods section.

We hope that you will find our revised manuscript suitable for publication.

**Below are point-by-point responses to reviewers' comments**

**Reviewer's Comments:**

**Reviewer #1 (Remarks to the Author)**

The author analysed 7691 invasive *Salmonella* isolates from 77 countries across 6 continents, covering a period of 70 years. This longitudinal genomic epidemiology study is the largest invasive *S. enterica* isolates ever performed at a global scale.

They observed an increasing iNTS serovar diversity over time, and an obvious shift in dominant serovars and STs worldwide across 6 continents. The majority of invasive *S. enterica* isolates in this study were susceptible to beta-lactams, and the resistant rate was higher than that in a previous study at a global scale.

They also observed increasing trends in clinically critical antibiotics, including ESBLs, including 3GCs (ceftriaxone, cefotaxime, and ceftazidime), 4GC (cefepime), ciprofloxacin, and azithromycin worldwide during 1953-2024, except for Oceania and South America.

This study highlights the value of integrating genomic surveillance for decision-makers in formulating effective AMR control and prevention of the dissemination of high-risk MDR and extensively drug-resistant clones.

Response: We express our sincere thanks to the reviewer for the very thoughtful and positive comments with great detail, which can help us further improve the quality of this work.

**Minor comments;**

1) Line 553-554, “ResFinder version 4.1 (coverage ≥ 90% and identity ≥ 95%), PlasmidFinder version 2.1 (coverage ≥ 60% and identity ≥ 90%),” What were the reason behind choosing the coverage of 90% for ResFinder and coverage of 60% for PlasmidFinder? In principle, ResFinder uses sequence alignment in gene level. Meanwhile, PlasmidFinder uses sequence alignment in replicon level which is around 100-300 bps. I think for PlasmidFinder the coverage should be high (more than 90%) as you target small sequence of replicon.

Response: Thanks for your suggestions. We have re-analyzed all plasmid replicon data using PlasmidFinder with a more stringent coverage threshold of ≥ 90% (while maintaining identity ≥ 95%). We have updated the description and figures in the manuscript accordingly. The revised Figure 4 and Supplementary Figure 11 are shown below.

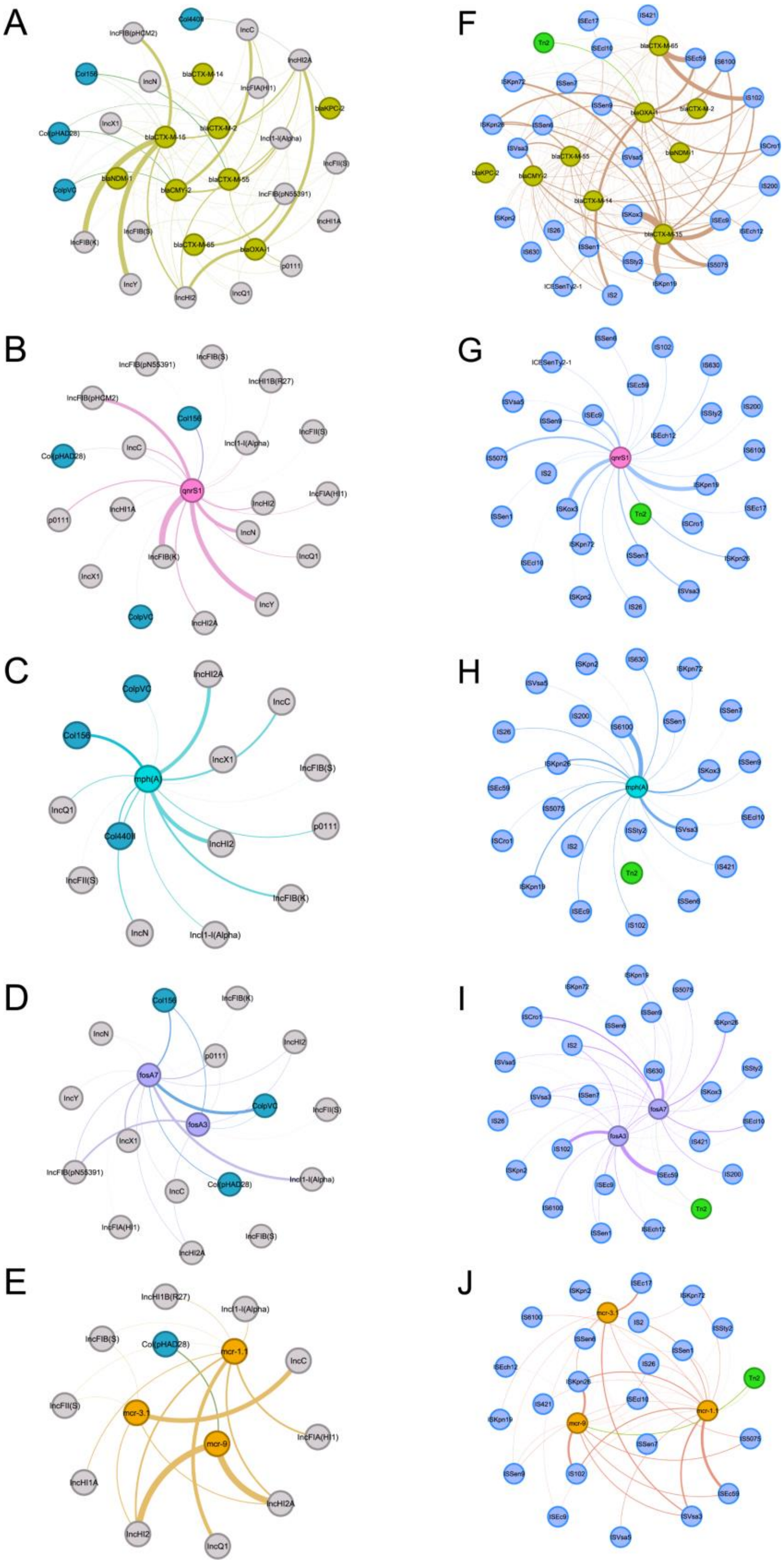


Figure 4. Correlation network between clinically critical ARGs and MGEs.

A-E, The co-occurrence network between plasmid replicons and 3GCs (A), ciprofloxacin (B), azithromycin (C), fosfomycin (D), and polymyxin (E) resistance genes, respectively. F-J, The co-occurrence network between MGEs and 3GCs (F), ciprofloxacin (G), azithromycin (H),

fosfomycin (I), and polymyxin (J) resistance genes, respectively.

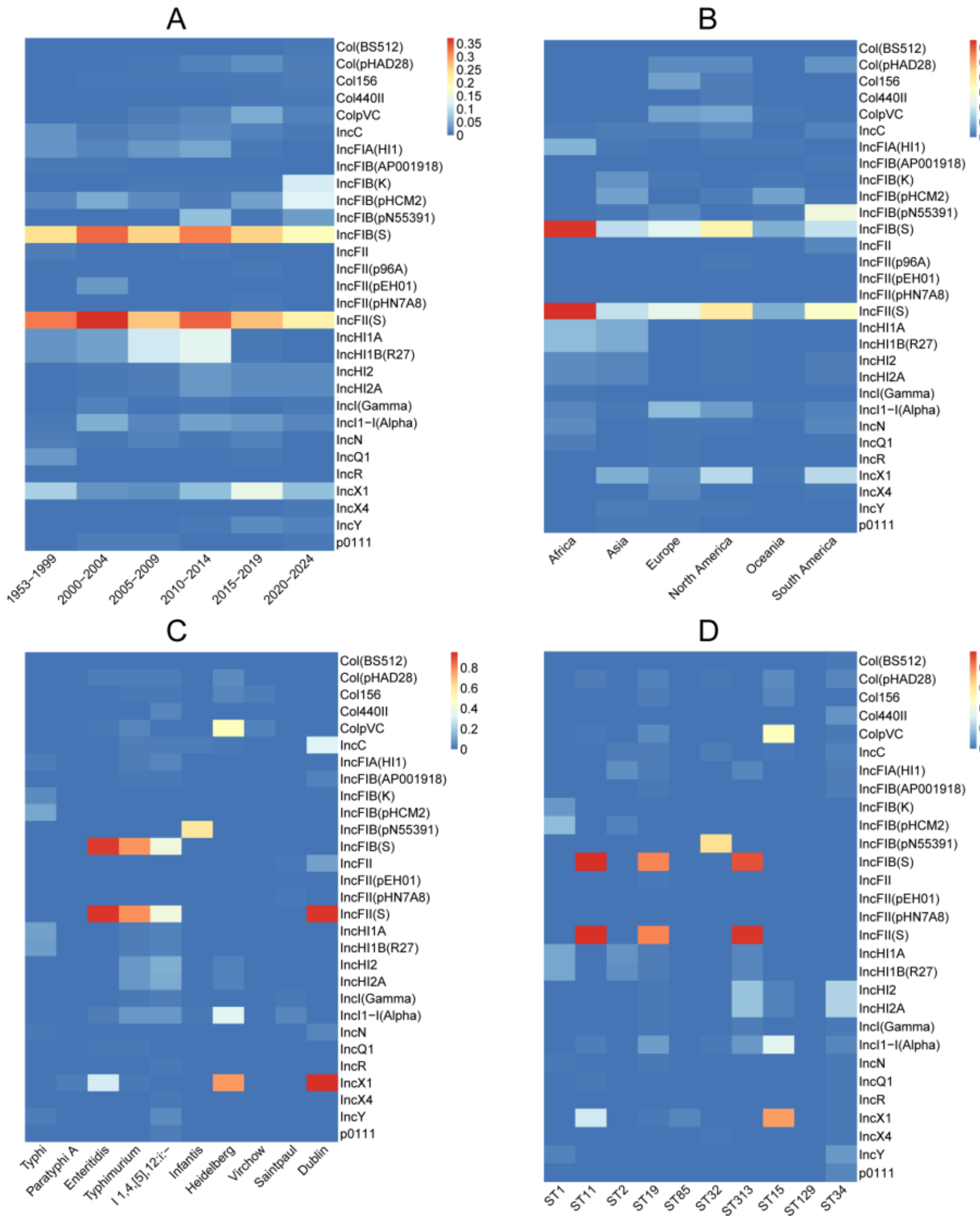


Supplementary Figure 11. Temporal dynamics and distribution characteristics of plasmids in invasive *Salmonella* derived from humans. A, Temporal dynamics of plasmids. B, Geographical distribution of plasmids in 6 continents. C, Distribution of plasmids in the top 10 serovars. D, Distribution of plasmids in the top 10 STs.

Line 378-398: A total of 67 different plasmid replicons were identified (Supplementary Tables 12). Some replicons, for example, IncFIB(K), IncFIB(pHCM2), and IncX1 present an obviously upward trend over time (Supplementary Figure 11A and Supplementary Table 13). IncFIB(S) and IncFII(S) were in a dominant position in Africa, and IncX1 was in a dominant position in Asia, North America, and South America (Supplementary Figure 11B). In different serovars, IncFIB(S) and IncFII(S) had a dominant proportion in *S*. Enteritidis, *S*. Typhimurium,

and *S*. I 1,4,[5],12:i:-. In *S*. Dublin, IncFII(S) and IncX1 had an advantageous proportion (Supplementary Figure 11C). In different STs, IncFIB(S) and IncFII(S) were in an advantageous position in *S*. Enteritidis ST11, *S*. Typhimurium ST313, and *S*. Typhimurium ST19 (Supplementary Figure 11D).

A total of 170 determined MGEs were identified (Supplementary Table 14). Globally, IS*Ec9*, IS*Kpn19*, and IS*Sen1* show an upward trend (Supplementary Figure 12A and Supplementary Table 15). IS*26* and IS*6100* were mainly detected in Europe, IS*Sen6* was mainly detected in Asia, Oceania, and Europe, and IS*Ecl10* and IS*Kpn2* were mainly detected in Africa and Europe (Supplementary Figure 12B). In *S*. Typhi, IS*Sen6* and IS*Sty2* had an advantage proportion, and in *S*. Paratyphi A, IS*Ech12*, IS*Sen6*, and IS*Sty2* had an advantage proportion. IS*Sen1* and IS*Sen7*, IS*Ecl10* were mainly detected in NTS, for example, *S*. Enteritidis, *S*. Typhimurium, *S*. Saintpaul, and *S*. Dublin (Supplementary Figure 12C). In different STs, IS*26* was dominantly detected in *S*. Typhimurium ST313 and ST34, and IS*Ech12* was dominantly detected in *S*. Paratyphi A ST85, *S*. Infantis ST32, and *S*. Paratyphi A ST129 (Supplementary Figure 12D).

2). The authors used both ResFinder and ABRicate for AMR gene determination. Why did you use both AMR tools? Which results did you use ResFinder and which ones you used ABRicate and why?

Response: We are sorry for the misleading description and have modified these sentences.

In this work, ResFinder was used only for AMR gene detection, while ABRicate was used only for VF detection. We did not use ABRicate for AMR genes. The sentence in the Materials and Methods section has been revised, as follows:

Lines 569-578: ResFinder version 4.1 (52, 53) (coverage ≥ 90% and identity ≥ 95%) were used to identify acquired AMR genes and chromosomal gene mutations mediating AMR. PlasmidFinder version 2.1 (62) (coverage ≥ 90% and identity ≥ 95%) was used to identify plasmid replicons. MobileElementFinder version 1.0.5 (63) (coverage ≥ 90% and identity ≥ 90%) was used to identify MGEs. SPIFinder version 2.0 (64) (coverage ≥ 60% and identity ≥ 95%) was used to identify *Salmonella* pathogenic islands (SPIs). ABRicate version 1.0.1 was used to identify VFs with default parameters (coverage ≥ 80% and identity ≥ 80%) (21).

3). The authors used Gubbins to remove recombination sites.

It would be beneficial for readers to specify the number of total prior and after recombination removal.

Response: Thanks for your suggestions. We used Gubbins to identify and remove recombinant regions prior to phylogenetic reconstruction. For the ST34 isolates, a total of 2,535 SNPs were detected in the core genome alignment before recombination removal, and 2,527 SNPs remained after filtering recombinant regions. For the ST32 isolates, 3,039 SNPs were identified before recombination removal, and 2,640 SNPs were retained after recombination filtering. Then, we reconstructed the phylogenetic tree based on the recombination-filtered SNPs. We have revised and supplemented the Methods and Results sections of the manuscript.

Line 589-592: For the ST34 isolates, a total of 2,535 SNPs were detected in the core genome alignment before recombination removal, and 2,527 SNPs remained after filtering recombinant regions. For the ST32 isolates, 3,039 SNPs were identified before recombination removal, and 2,640 SNPs were retained after recombination filtering.

Lines 431-442: Based on 2,640 SNPs, global invasive ST32 can be divided into 4 clades (Supplementary Figure 14B). Clade 1 is composed of strains mainly from an African country (South Africa). Clade 2 is mainly composed of North American countries (Canada). Clade 3 is further divided into 3.1 and 3.2, with 3.1 mainly composed of strains from the South American country (Peru) and 3.2 mainly composed of strains from the South American (Peru) and North American countries (Canada and USA) (Supplementary Figure 14B). The *aadA1*, *aph(3')-Ia*, *dfrA14*, *sul1*, *tet(A)*, and *gyrA* p.D87Y genes were mainly detected in clade 3, *aac(3)-IV*, *aph(4)-Ia*, *floR*, *fosA3*, and $bla_{CTX\text{-}M\text{-}65}$ were detected in clade 3.2 (Supplementary Figure 14B). In addition, *floR* and *tet(A)* were detected in clade 1. The distribution of VFs also varies among different clades. Adherence-coding genes *faeD*/*E* and nutritional/metabolic factor-coding genes *fyuA*, *irp1*/*2*, *ybtA*/*X* were mainly detected in clade 3 (Supplementary Figure 14B).

The revised phylogenetic tree is shown in the figure below.

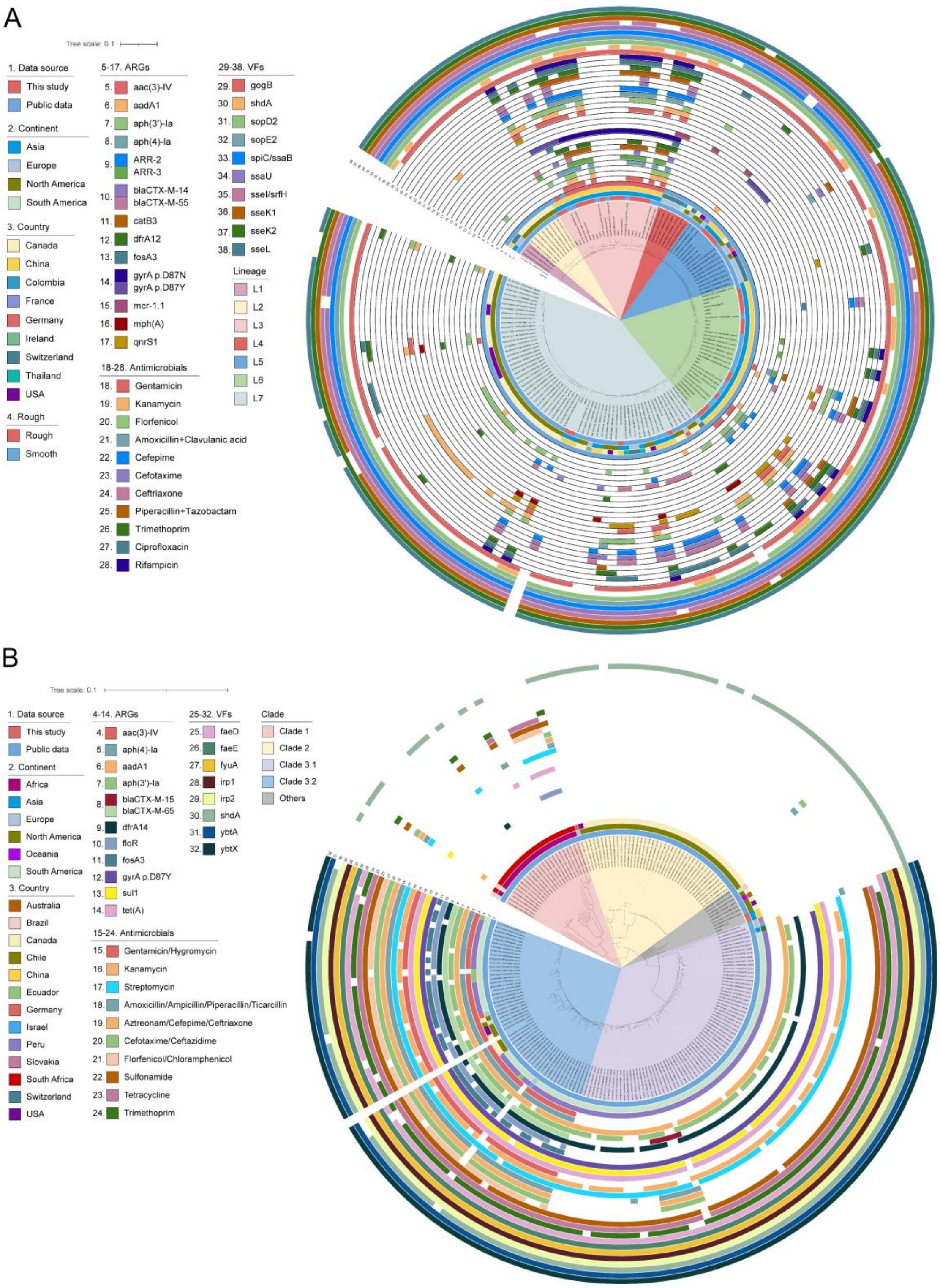


Supplementary Figure 14. Phylogenetic analysis of dominant iNTS ST (ST34 and ST32) causing human invasive infections.

A, Phylogenetic analysis of invasive ST34 uncovered differences in antimicrobial resistance and virulence factors among different lineages. A phylogenetic tree based on the maximum likelihood estimation (MLE) method is built with 2,527 SNPs in the core genomes of 149

genomes. *S*. Typhimurium str. LT2 (accession number: AE006468) was selected as the reference genome. From the inner to outer circles are data source, continent, country, rough, ARGs, antimicrobials, VFs, and lineages, respectively. B, Phylogenetic analysis of invasive ST32 revealed differences in antimicrobial resistance and virulence factors among different clades. A phylogenetic tree based on the maximum likelihood estimation (MLE) method is built with 2,640 SNPs in the core genomes of 198 genomes. *S*. Infantis ST32 (accession number: GCA_001931595.1) was selected as the reference genome. From the inner to outer circles are data source, continent, country, ARGs, antimicrobials, VFs, and clades, respectively.

All authors appreciate the reviewers again for these invaluable comments and suggestions. We will be happy to edit the text further based on helpful comments from the reviewers.

**Reviewer #2 (Remarks to the Author):**

This manuscript describes a large population genomic study (7,691 isolates) of *Salmonella enterica* susbsp. *enterica* to study prevalence and spatiotemporal trends of antimicrobial resistance within this subspecies. The spatiotemporal aspect of this study makes this an interesting topic of importance to a wide audience. There are several aspects of the manuscript that have to be addressed that would improve the readability and correctness.

Response: We thank the reviewer for the useful comments and valuable suggestions, which can help us further improve the quality of this work.

Throughout the article the authors suggest they are presenting data on antibiotic resistance, susceptibility and non-susceptibility; this suggest an understanding of the resistance phenotype of the isolates; this is not the case, everything in this article is based on genomic data. The manuscript should be revised to make it abundantly clear that it is not the 'prevalence and spatiotemporal trends of antimicrobial resistance' that is presented, but the prevalence and spatiotemporal trends of antimicrobial resistance conferring genes and mutations.

Response: We agree with Reviewer #2 that the resistance phenotype of the isolates was predicted based on WGS using ResFinder, and the prevalence and spatiotemporal trends of antimicrobial resistance phenotype were predicted based on the presence or absence of AMR markers. We have added the relevant description to the method section to make it more readable and friendly for the readers.

Line 571-572: The resistance phenotypes of the isolates were predicted based on AMR genotypes.

Given the format/flow of the article (introduction, results, discussion, materials and methods), it would be good to introduce some of the methodology in the results section to give the reader the information to understand the results presented. For example, what were the inclusion criteria for genomes? Given the fact that data for 548,830 genomes was available to the authors, this may explain to the reader why only a small fraction was used and that a lot of data from human cases of the USA is not included. Include which genotyping methods, MLST or wgMLST schemes were used.

Response: We thank the reviewer for this constructive suggestion.

- First, we have added some of the methodology in the results section "Construction of a global invasive *Salmonella* genomic dataset".

Line 135-144: In this study, the invasive *Salmonella* data used here included two parts. One part is from the retrospective genomic epidemiological study in China, and another part is from publicly available *Salmonella* genomes in the National Center of Biotechnology Information (NCBI). A total of 585,220 publicly available *Salmonella* genomes were downloaded from the National Center for Biotechnology Information (as of February 14, 2025), after screening by using SeqSero2, STSTR, and MLST, as well as clear metadata information. A total of 260,195 high-quality genomes were used for the following analysis. Among them, 117,461 strains were isolated from humans, 6,781 strains were isolated from blood, urine, and others from extra-intestinal infection sites, and were further selected for AMR analysis.

- Second, the vast majority of public data—including many human isolates from the USA—were not included in this study due to the incomplete metadata (genomes with clear metadata, including collection time, isolation source, and locations) or failed typing predictions (SeqSero2, STSTR, and MLST).
- Third, regarding genotyping methods: we used multilocus sequence typing (MLST) based on seven housekeeping genes, the Paratype scheme for *Salmonella* Paratyphi A (Tanmoy et al., Nat Commun 2022), and the Genotyphi scheme for *Salmonella* Typhi (Wong et al., Nat Commun 2016).

Lines 578-581: The genotypes for *S*. Paratyphi A were determined using Paratype version 1.1 (44) with the following parameters: --read_cutoff 0.75, --phrd_cutoff 20, and --mapq_cutoff 20. The genotypes for *S*. Typhi were determined using GenoTyphi version 2.10 (65) with default parameters (--species typhi).

When STs are mentioned, always include the serovar name associated with the ST (if possible) to increase readability.

Response: Thanks for your suggestions. We have revised the manuscript accordingly to make it more readable and friendly for the readers.

The title does not seem to reflect the subject of the manuscript; it suggests the emergence of a (single) ciprofloxacin and ceftriaxone-resistant clone. Please revise the title.

Response: Thanks for your suggestions. We have revised the title of this manuscript. The new title: Global genomic surveillance uncovers emergence and spatiotemporal patterns of World Health Organization priority antibiotic resistance in invasive *Salmonella*.

All authors appreciate the reviewers again for these invaluable comments and suggestions. We will be happy to edit the text further based on helpful comments from the reviewers.
