# Supplementary material for: Global genomic surveillance uncovers emergence and spatiotemporal patterns of World Health Organization priority antibiotic resistance in invasive Salmonella
Source: Nat Commun. 2026 Aug 18;17:9871. doi: 10.1038/s41467-026-76354-1 (PMC13578455; doi:10.1038/s41467-026-76354-1)
Supplement: Supplementary file 4 [file 41467_2026_76354_MOESM1_ESM.docx]

**Global genomic surveillance uncovers emergence and spatiotemporal patterns of World Health Organization priority antibiotic resistance in invasive *Salmonella***

Yuhang Pei^1,2,#^, Ziru Yang^1^^,2,#^, Xiaolu Pang^3^, Wufuer. Xiayidan^4^, Meilian Huang^5^, Jinhua Meng^6^, Yufeng Qiu^7^, Mingliu Wang^8^, Hongxia Yang^9^, Chunmei Jing^10^, Mei Zeng^11^, Yue Liu^12^, Wenqing Wang^13^, Yibin Zhou^14^, Sufang Ma^15^, Na Lyu^15^, Mengqi Qu^1^, Chaochao Zhao^1^, Jiangtao Wang^1^, Jiale Chen^1^, Baoli Zhu^15,16^, George F. Gao^15,16,17^, Xuebin Xu^12,*^, Yanan Wang^1,2,15,*^

^1^International Joint Research Center of National Animal Immunology, College of Veterinary Medicine, Henan Agricultural University, Zhengzhou 450046, China.

^2^Longhu Laboratory of Advanced Immunology, Zhengzhou 450046, China.

^3^Department of Clinical Laboratory, The Fourth People’s Hospital of Nanning, Nanning 530023, China.

^4^Department of Microbiology, Xinjiang Uygur Autonomous Region Center for Disease Control and Prevention, Urumqi 830002, China.

^5^Department of Laboratory Medicine, Children’s Hospital of Fudan University (Xiamen Branch), Xiamen Children’s Hospital, Xiamen 361000, China.

^6^Center of Clinical Medical Laboratory, Shanxi Children’s Hospital, Taiyuan 30013, China.

^7^Fujian Center for Disease Control and Prevention, Fujian Provincial Key Laboratory of Zoonosis Disease Research, Fuzhou 350001, China.

^8^Guangxi Zhuang Autonomous Region Center for Disease Control and Prevention, Nanning, 530028, China.

^9^Shanxi Center for Disease Control and Prevention, Taiyuan 030012, China.

^10^Children’s Hospital of Chongqing Medical University, Chongqing 401122, China.

^11^Department of Infectious Diseases, Children's Hospital of Fudan University, Shanghai 201102, China.

^12^Division of Pathogen Testing and Analysis, Shanghai Municipal Center for Disease Control and Prevention, Shanghai 201107, China.

^13^Department of Microbiology, Shanghai Pudong New Area Center for Disease Control and Prevention (Shanghai Pudong New Area Health Supervision Institute), Shanghai 200136, China.

^14^Department of Infectious Disease Control, Center for Disease Control and Prevention of Minhang District, Shanghai 201108, China.

^1^^5^Beijing Key Laboratory of Antimicrobial-Resistant Pathogen Microbiology and AI-Empowered Containment, Institute of Microbiology, Chinese Academy of Sciences, Beijing 100101, China.

^16^University of Chinese Academy of Sciences, Beijing 100049, China.

^17^Chinese Center for Disease Control and Prevention (China CDC), Beijing 102206, China.

^#^These authors contributed equally to this work.

*E-mail: (YNW) [wangyanan1001@henau.edu.cn](mailto:wangyanan1001@henau.edu.cn), (XBX) xuxuebin@scdc.sh.cn

**Supplementary Figure 1. Summary of the six continents of invasive *Salmonella* causing human infections.**

Distribution of different countries (A) and year groups (B). Different colors represent distinct categories, with full color labels provided in the legend of each panel.

**Supplementary Figure 2. Distribution of the top 15 serovars of invasive *Salmonella* derived from humans on six continents.**

A, Proportion of top 15 serovars in six continents. B, Temporal trends of the top 15 serovars in six continents. Different colors represent distinct categories, with full color labels provided in the legend of each panel.

**Supplementary Figure 3. Distribution characteristics of different genotypes in *S*. Typhi.**

A, Distribution of different genotypes in 5 continents. B, Distribution of different subtypes in 5 continents. C, Distribution of different genotypes in different countries. D, Distribution of different subtypes in different countries. E, Temporal dynamics of different genotypes. F, Temporal dynamics of different subtypes. Different colors represent distinct categories, with full color labels provided in the legend of each panel.

**Supplementary Figure 4. Distribution characteristics of different genotypes in *S*. Paratyphi A.**

A, Distribution of different genotypes in 4 continents. B, Distribution of different subtypes in 6 continents. C, Distribution of different genotypes in different countries. D, Distribution of different subtypes in different countries. E, Temporal dynamics of different genotypes. F, Temporal dynamics of different subtypes. Different colors represent distinct categories, with full color labels provided in the legend of each panel.

**Supplementary Figure 5. The minimum spanning tree (MST) of invasive *Salmonella* derived from humans based on the multi-locus sequence typing.**

A, Year group. B, Continent. Different colors represent distinct categories, with full color labels provided in the legend of each panel.

**Supplementary Figure 6. Distribution of the top 15 STs of invasive *Salmonella* derived from humans across six continents.**

A, Proportion of top 15 STs in six continents. B, Temporal trends of the top 15 STs in six continents. Different colors represent distinct categories, with full color labels provided in the legend of each panel.

**Supplementary Figure 7. Temporal dynamics of clinically critical ARGs in invasive *Salmonella* derived from humans in the top 16 serovars.**

Different colors represent distinct ARG classes, with full color labels provided in the legend.

**Supplementary Figure 8. Temporal dynamics of clinically critical ARGs in invasive *Salmonella* derived from humans in the top 16 STs.**

Different colors represent distinct ARG classes, with full color labels provided in the legend.

**Supplementary Figure 9. Distribution characteristics of clinically critical ARGs in invasive *Salmonella* derived from humans.**

A, Geographical distribution of ARGs in six continents. B, Distribution of ARGs in the top eight serovars. Different colors represent distinct ARG classes, with full color labels provided in the legend.

**Supplementary Figure 10. Temporal trends of the number of antimicrobial resistance genes (ARGs) in invasive *Salmonella* derived from humans.**

A, Temporal trends of ARG numbers per genome in global isolates. Comparisons between different year groups were performed using the Kruskal-Wallis test. The “*” represents *P* values. *: *P*<0.05; **: *P*< 0.01; ***: *P*< 0.001; ****: *P*< 0.0001. No significant differences were not shown. Data are presented as mean values with SEM. Global: n = 487, 334, 762, 1,738, 3,094, 1,276. Asia: n = 287, 196, 483, 663, 961, 598. North America: n = 4, 6, 63, 352, 1,485, 88. Europe: n = 6, 4, 24, 35, 53, 7. Oceania: n = 6, 14, 133, 415, 536. South America: n = 50, 17, 42, 194, 105, 30. Africa: n = 134, 110, 136, 361, 75, 17. B, Temporal trends of ARG numbers per genome in different serovars belonged to the top 20. Typhi: n = 298, 184, 413, 714, 1,059, 345. Choleraesuis: n = 6, 3, 31, 37, 13, 2. Dublin: n = 42, 5, 7, 23, 29. Typhimurium: n = 21, 63, 54, 197, 149, 105. Panama: n = 9, 3, 8, 17, 46. Saintpaul: n = 6, 20, 83. Newport: n = 16, 34, 12. Javiana: n = 7, 35, 9. Paratyphi A: n = 7, 52, 20, 224, 118. Enteritidis: n = 71, 56, 102, 337, 690, 168. Virchow: n = 3, 3, 9, 103. I 1,4,[5],12:i:-: n = 24, 64, 80, 62. Infantis: n = 18, 127, 62, 11. Heidelberg: n = 3, 54, 104. C, Temporal trends of ARG numbers per genome in different STs belonged to the top 20. ST1: n = 31, 76, 145, 509, 775, 299. ST2: n = 263, 107, 214, 255, 276, 45. ST68: n = 4, 3, 30, 30, 8. ST313: n = 16, 61, 19, 61, 17, 6. ST10: n = 21, 4, 4, 22, 29. ST198: n = 10, 12, 13, 10. ST50: n = 4, 6, 81. ST48: n= 9, 3, 10, 15, 46. ST19: n = 7, 49, 156, 134, 113. ST16: n = 6, 6, 103. ST32: n = 14, 115, 58, 11. ST15: n = 3, 53, 104. ST34: n = 8, 40, 63, 38. ST129: n = 4, 4, 80, 70.

**Supplementary Figure 11. Temporal dynamics and distribution characteristics of plasmid replicons in invasive *Salmonella* derived from humans.**

A, Temporal dynamics of plasmids. B, Geographical distribution of plasmids in 6 continents. C, Distribution of plasmid replicons in the top 10 serovars. D, Distribution of plasmid replicons in the top 10 STs.

**Supplementary Figure 12. Temporal dynamics and distribution characteristics of MGEs in invasive *Salmonella* derived from humans.**

A, Temporal dynamics of MGEs. B, Geographical distribution of MGEs in 6 continents. C, Distribution of MGEs in the top 10 serovars. D, Distribution of MGEs in the top 10 STs.

**Supplementary Figure 13. Temporal dynamics and distribution of virulence factors (VFs) of invasive *Salmonella* derived from humans.**

A, Temporal dynamics of VFs. B, Geographical distribution of VFs. C, Distribution of VFs in the top 10 serovars. D, Distribution of VFs in top 10 STs. Different colors represent distinct virulence factor classes.

**Supplementary Figure 14. Phylogenetic analysis of dominant iNTS ST (ST34 and ST32) causing human invasive infections.**

A, Phylogenetic analysis of invasive ST34 uncovered differences in antimicrobial resistance and virulence factors among different lineages. A phylogenetic tree based on the maximum likelihood estimation (MLE) method is built with 2,527 SNPs in the core genomes of 149 genomes. *S*. Typhimurium str. LT2 (accession number: AE006468) was selected as the reference genome. From the inner to outer circles are data source, continent, country, rough, ARGs, antimicrobials, VFs, and lineages, respectively. B, Phylogenetic analysis of invasive ST32 revealed differences in antimicrobial resistance and virulence factors among different clades. A phylogenetic tree based on the maximum likelihood estimation (MLE) method is built with 2,640 SNPs in the core genomes of 198 genomes. *S*. Infantis ST32 (accession number: GCA_001931595.1) was selected as the reference genome. From the inner to outer circles are data source, continent, country, ARGs, antimicrobials, VFs, and clades, respectively. Different colors represent distinct categories, with full color labels provided in the legend of each panel.
